# Stroma-specific gene expression signature identifies prostate cancer subtype with high recurrence risk

**DOI:** 10.1038/s41698-024-00540-x

**Published:** 2024-02-23

**Authors:** Martin Rasmussen, Jacob Fredsøe, Paul Vinu Salachan, Marcus Pii Lunau Blanke, Stine Hesselby Larsen, Benedicte Parm Ulhøi, Jørgen Bjerggaard Jensen, Michael Borre, Karina Dalsgaard Sørensen

**Affiliations:** 1 Department of Molecular Medicine, Aarhus University Hospital (AUH), Aarhus, Denmark; 2 Department of Clinical Medicine, Aarhus University, Aarhus, Denmark; 3 Department of Pathology, Aarhus University Hospital (AUH), Aarhus, Denmark; 4 Department of Urology, Gødstrup Hospital, Herning, Denmark; 5 Department of Urology, Aarhus University Hospital (AUH), Aarhus, Denmark

**Keywords:** Prostate cancer, Molecular medicine, Cancer microenvironment, Tumour biomarkers

## Abstract

Current prognostic tools cannot clearly distinguish indolent and aggressive prostate cancer (PC). We hypothesized that analyzing individual contributions of epithelial and stromal components in localized PC (LPC) could improve risk stratification, as stromal subtypes may have been overlooked due to the emphasis on malignant epithelial cells. Hence, we derived molecular subtypes of PC using gene expression analysis of LPC samples from prostatectomy patients (cohort 1, n = 127) and validated these subtypes in two independent prostatectomy cohorts (cohort 2, n = 406, cohort 3, n = 126). Stroma and epithelium-specific signatures were established from laser-capture microdissection data and non-negative matrix factorization was used to identify subtypes based on these signatures. Subtypes were functionally characterized by gene set and cell type enrichment analyses, and survival analysis was conducted. Three epithelial (E1-E3) and three stromal (S1-S3) PC subtypes were identified. While subtyping based on epithelial signatures showed inconsistent associations to biochemical recurrence (BCR), subtyping by stromal signatures was significantly associated with BCR in all three cohorts, with subtype S3 indicating high BCR risk. Subtype S3 exhibited distinct features, including significantly decreased cell-polarity and myogenesis, significantly increased infiltration of M2-polarized macrophages and CD8 + T-cells compared to subtype S1. For patients clinically classified as CAPRA-S intermediate risk, S3 improved prediction of BCR. This study demonstrates the potential of stromal signatures in identification of clinically relevant PC subtypes, and further indicated that stromal characterization may enhance risk stratification in LPC and may be particularly promising in cases with high prognostic ambiguity based on clinical parameters.

## Introduction

Prostate cancer (PC) is the third leading cause of cancer-associated mortality and the most commonly diagnosed non-skin cancer among men in the west^1^. While indolent PC can often be managed by active surveillance, early stage aggressive PC requires active treatment by radical prostatectomy (RP) or radiation therapy to avoid metastatic spread^2^. Several prognostic nomograms based on clinical variables, e.g., Gleason Grade, tumor stage and serum prostate specific antigen (PSA), have been developed in an attempt to stratify localized PC (LPC) patients into low, intermediate or high-risk groups^2,3^. However, models based exclusively on clinical parameters offer limited accuracy and cannot readily distinguish between aggressive and indolent PC at the early disease stage. This is evident by the overtreatment of indolent PC that can lead to unnecessary side effects^4^, and undertreatment or delayed treatment of potentially aggressive PC resulting in ~30% of patients treated by RP experiencing biochemical recurrence (BCR) within a 10-year period^5,6^. Thus, there is a need for a better risk stratification tool to improve decision-making in LPC.

While PC originates in the prostate epithelium, the stromal composition has become increasingly recognized as an important contributor to disease initiation, progression, and response to treatment^7^. The environment of cells around the PC cells, known as the tumor microenvironment (TME), can act reciprocally with the cancer cells to influence aggressiveness^8^. Various characteristics of the TME, such as infiltration of specific immune cell types and transformation of resident stromal cells, have been associated with recurrence and metastatic spread across cancer types, including PC^9–11^. Previous studies have reported high abundance of cancer-associated fibroblasts, changes to the extracellular matrix, and increased vessel formation as indications of aggressive PC^12,13^. Increases in specialized macrophages (M2-polarized macrophages) and regulatory T-cells have also been associated to invasion and higher risk of metastatic spread in PC^14,15^, while other subsets of T-cells (e.g., CD8 + T-cells) may affect PC progression as suggested by their association to poor metastasis-free survival^16^. Hence, the specific cell type composition of the TME as well as the interactions between different cell types may reflect PC aggressiveness, and further investigations are required to fully understand this.

For some cancers, e.g., pancreatic cancer and colorectal cancer, it has been suggested that patients can be grouped into several subtypes with distinct prognosis based on their TME composition, including differences in the presence of certain immune and stromal cell types (e.g., fibroblasts, neutrophils and CD8 + T-cells) and in certain cancer cell signaling pathways (e.g., NOTCH1 signaling)^17,18^. However, limited attention has been given to the importance of TME subtypes in PC biology and progression^19^. Thus, we aimed to investigate if separate analysis of epithelium- and stroma-specific gene expression patterns could identify novel and clinically-relevant subtypes of PC.

We used three large RP cohorts to identify and validate prognostic subtypes of LPC. Based on unsupervised clustering using epithelium or stroma-specific gene expression signatures from prostate samples, we identified three epithelial and three stromal subtypes of early-stage, clinically-localized PC. Both epithelial and stromal subtypes were characterized using clinicopathological characteristics (e.g., Gleason Grade, T-stage), gene set enrichment analysis, and stromal and immune cell infiltration patterns. Lastly, we used epithelial and stromal subtypes to identify high-risk subsets of PC patients and to improve the accuracy of risk prediction for patients classified as intermediate risk based on clinical factors (CAPRA-S nomogram).

## Results

### Definition of epithelium- and stroma-specific gene expression signatures and derivation of epithelial and stromal subtypes

**Fig. 1:**
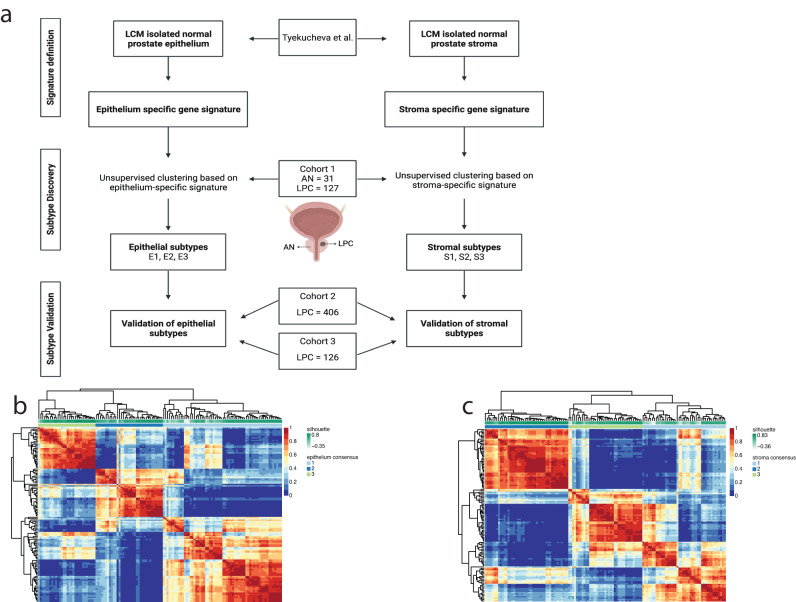
Workflow for subtype discovery. **a** Workflow and patient samples across cohorts. Heatmap of the three-group consensus cluster for the (**b**) epithelium-specific gene expression signature or (**c**) stroma-specific gene expression signature.

We hypothesized that clinically relevant subtypes of PC could be identified by separating the contribution of stroma (TME) from that of the epithelium (PC cells). To investigate this, we searched for genes that were specifically expressed in either prostate epithelium or prostate stroma using data from laser capture microdissected healthy prostate epithelium and matched healthy stroma samples from Tyekucheva et al.^19^. (Fig. 1a). Identification of genes with expression specific for epithelium or stroma, and involved in Gene Ontology pathways related to epithelium- or stroma-specific functions, resulted in a prostate epithelium-specific gene signature (n = 86 genes, Supplementary Table 1) and a prostate stroma-specific gene signature (n = 88 genes, Supplementary Table 1).

First, to test the significance of the epithelium- and stroma-specific gene signatures, we generated total RNA sequencing data from 127 LPC tumor samples. To ensure the cohort was representative for PC, we further included 31 adjacent normal samples (AN) (cohort 1, discovery), allowing us to identify well-known PC-associated gene differences between LPC and AN. An overview of the study design is given in Fig. 1a. Unsupervised clustering, using Euclidean distance and complete clustering-linkage based on the 500 most variably expressed genes, resulted in two distinct clusters, with all but one AN sample in one cluster and the vast majority of LPC samples in the other cluster (Supplementary Fig. 1a). Differential gene expression (DGE) analysis identified 4954 downregulated and 6246 upregulated genes in LPC compared to AN samples (BH-adj. *p* < 0.05). The most significantly upregulated genes in LPC samples were PC-associated genes (e.g., *DLX1*, *HPN*, and *SIM2*^20–22^), and well known PC-associated oncogenes such as *ERG*, *MYC*, and *KLK3*^23^ were similarly upregulated in LPC samples (Supplementary Fig. 1b), supporting the validity of our data and indicating that it is a representative RP patient cohort.

Next, we used the epithelium and stroma-specific gene signatures to sub-classify the LPC tumor samples from cohort 1 based solely on their expression of epithelium or stroma specific-genes (Fig. 1a). Using non-negative matrix factorization (NMF) and consensus clustering, we evaluated a range of potential subtype partitions (2–8 clusters) and based on cophenetic and silhouette scores identified the optimal number of partitions to use for the final analysis. Specifically, we classified samples into three PC subtypes based on the epithelial (E) gene expression signature (E1, E2 and E3; n = 61, 34, and 29 patients, respectively; Fig. 1b; Supplementary Fig. 2a) and three PC subtypes based on the stromal (S) gene expression signature (S1, S2 and S3; n = 44, 43, and 37 patients, respectively; Fig. 1c; Supplementary Fig. 2b). Subtypes were subsequently validated by NMF and consensus clustering also in the external, publicly available cohort 2 (TCGA) and cohort 3 (MSKCC) (Fig. 1a). The overlap of epithelial (E1–E3) and stromal (S1–S3) subtypes in cohorts 1–3 is given in Supplementary Fig. 3, and clinicopathological characteristics for cohorts 1–3 are given in Table 1.

**Table 1:** Clinicopathological characteristics of patient sample sets

	Cohort 1 Discovery (total RNA-seq)	Cohort 2 Validation (TCGA-PRAD)	Cohort 3 Validation (MSKCC)
	N = 127	N = 406	N = 126
Age (years)			
Median (range)	65 (45–78)	61 (41–78)	58 (37–72)
Pre-RP PSA (ng/mL)			
Median (range)	10.6 (2.0–193)	7.5 (0.8–107)	5.9 (1.2–46.4)
Unknown, n (%)	14 (11.0%)	15 (3.7%)	0 (0.0%)
ISUP Grade Group			
1	13 (10.24%)	39 (9.6%)	40 (31.7%)
2	68 (53.5%)	117 (28.8%)	52 (41.3%)
3	22 (17.3%)	78 (19.2%)	20 (15.9%)
4–5	23 (18.1%)	42 (36.4%)	14 (11.1%)
Unknown, n (%)	1 (0.8%)	0 (0.0%)	0 (0.0%)
pT stage			
pT2a-c	76 (59.8%)	150 (36.9%)	81 (64.3%)
pT3a	26 (20.5%)	131 (32.3%)	28 (22.2%)
pT3b-4	24 (18.9%)	119 (29.3%)	17 (13.5%)
Unknown, n (%)	1 (0.8%)	6 (1.5%)	0 (0.0%)
Margin status			
Negative	83 (65.4%)	260 (64.0%)	96 (76.2%)
Positive	41 (32.3%)	120 (29.6%)	30 (23.8%)
Unknown, n (%)	3 (2.4%)	26 (6.4%)	0 (0.0%)
Capra-S risk nomogram			
Low risk (0–2)	30 (23.6%)	95 (23.2%)	69 (54.8%)
Intermediate risk (3–5)	60 (47.2%)	144 (35.5%)	34 (27.0%)
High risk (≥6)	34 (26.8%)	122 (30.0%)	23 (18.3%)
Unknown, n (%)	3 (2.4%)	46 (11.3%)	0 (0.0%)
Biochemical Recurrence			
No	77 (60.5%)	348 (85.7%)	94 (74.6%)
Yes	50 (39.4%)	58 (14.3%)	32 (25.4%)
Follow-up length (Months)			
Median (range)	71 (2.9–204)	21.3 (3.1–151)	93.1 (10.4–223)
Survival status			
Alive	111 (87.4%)	NA	109 (76.5%)
Dead	16 (12.6%)	NA	17 (13.5%)

Data is in n (%) or median (range).

### Epithelial subtypes have growth and hormone regulation characteristics but heterogeneous prognostic potential across cohorts

We initially sought to characterize the three epithelial subtypes, E1, E2 and E3, identified above in cohort 1 (Fig. 1b) and validated in cohorts 2 and 3 (Supplementary Fig. 4a).

**Fig. 2:**
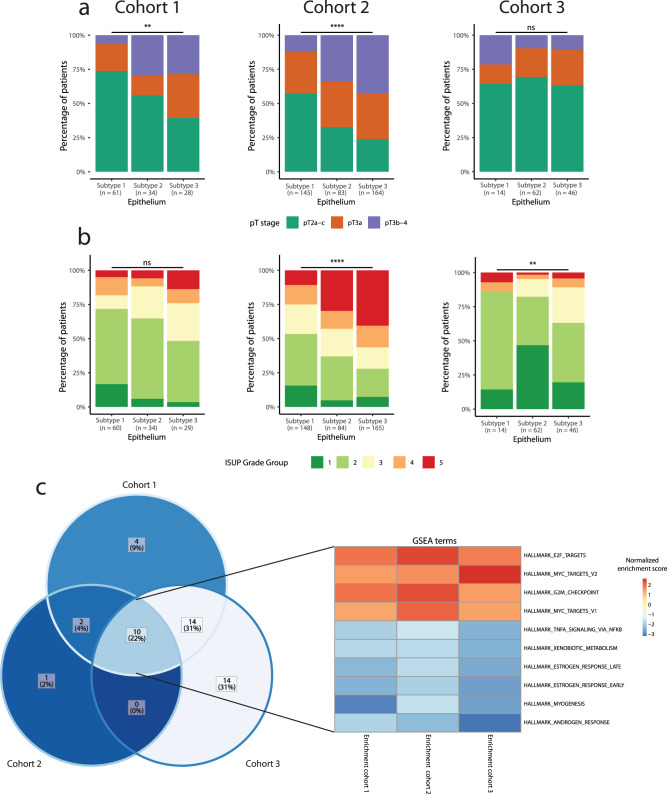
Epithelial subtype characteristics. Stacked bar plot in each cohort showing percentage of patients in each subtype according to (**a**) pathological T-stage, (**b**) 2014 ISUP Gleason grade. Fisher´s Exact test is used to determine significant differences in clinical characteristics. **c** Venn diagram and heatmap showing overlap of significantly enriched Hallmarks for GSEA of DGE between E1 and E3.

For each subtype, we analyzed the distribution of clinical variables known to be associated with adverse PC disease course. Subtype E3 showed an association to higher pT-stage and higher ISUP grade group, although this trend was not significant across all cohorts (Fig. 2a, b). Next, the subtype associated with the least (E1) and the most (E3) adverse clinicopathological characteristics, respectively, were further compared.

Gene set enrichment analysis (GSEA) based on genes differentially expressed between subtype E1 and E3 identified several Hallmark pathways important to PC biology (n = 30, 13, 38 in cohorts 1, 2, 3, respectively). Of these, 10 pathways were seen to be differentially expressed between E1 and E3 consistently in all 3 cohorts (encompassing 22% of unique enriched pathways across cohorts, *p* < 0.05, Fisher’s Exact Test; Fig. 2c), with crucial functions in cell growth (E2F and MYC target pathways) and a reduced hormone response (androgen and estrogen response pathways), indicating aggressive cancer growth and reduced sensitivity to hormone signaling in the E3 subtype. Overlap in pathways across cohorts likely indicate that the underlying disease biology differs between subtype E1 and E3.

Furthermore, cell-type enrichment analyses showed higher enrichment of M2-polarized macrophages and CD8 + T-cells in subtype E3 compared to E1 in both cohort 1 and cohort 2 (Supplementary Fig. 5). We and others have previously reported that high levels of infiltrating M2-polarized macrophages and CD8 + T-cells is associated with more aggressive PC^16,24,25^. No other cell type investigated (fibroblasts, smooth muscle cells, adipocytes, B-cells, CD4 + T-cells, dendritic cells, endothelial cells, eosinophils, epithelial cells, M1-polarized macrophages, mast cells, NK cells, and regulatory T-cells) showed significant difference in abundance between subtypes E1 and E3 that were consistent across both cohorts analyzed (Supplementary Fig. 5).

Lastly, prognostic evaluation showed significant association of subtype E3 with increased risk of post-operative BCR compared to E1 in cohort 1 (log-rank test, *p* = 0.032, Fig. 4a) and cohort 2 (log-rank test, *p* = 0.019, Fig. 4b) but not in cohort 3 (log-rank test, *p* = 0.433 Fig. 4c). These results were corroborated by univariate Cox regression analysis of time to BCR (Table 2). Subtype E3 was not significantly associated with BCR in any of the three cohorts when adjusting for clinical variables (CAPRA-S nomogram) in multivariate analysis (Table 3).

**Table 2:** Univariate Cox regression analysis of BCR-free survival and CAPRA-S risk groups, epithelial subtypes, and stromal subtypes

	Univariate cox regression														
	Cohort 1					Cohort 2					Cohort 3				
Variable	N	HR	95% CI	*p* value	C-index	N	HR	95% CI	*p* value	C-index	N	HR	95% CI	*p* value	C-index
CAPRA-S risk group					0.695					0.646					0.739
Low	29	-	-	-		94	-	-	-		69	-	-	-	
Intermediate	60	1.46	0.58–3.67	0.4		142	3.83	1.12–13.1	**0.032**		34	4.72	1.17–12.6	**0.002**	
High	32	5.61	2.26–13.9	**<0.001**		117	7.95	2.42–26.2	**<0.001**		19	7.29	2.63–20.2	**<0.001**	
Epithelium					0.589					0.597					0.540
Subtype 1	61	-	-	-		148	-	-	-		14	-	-	-	
Subtype 3	29	2.09	1.03–4.22	**0.041**		165	2.25	1.12–4.52	**0.023**		46	1.60	0.44–5.77	0.5	
Stroma					0.611					0.583					0.638
Subtype 1	44	-	-	-		198	-	-	-		54	-	-	-	
Subtype 3	37	3.21	1.48–6.94	**0.003**		162	2.14	1.18–3.87	**0.012**		18	3.42	1.12–9.47	**0.018**	

Significant *p* values (*p* < 0.05) are highlighted.

*HR* hazard ratio, *CI* confidence interval, *C-index* Harrell’s concordance index.

**Table 3:** Multivariate Cox regression analysis of BCR-free survival and CAPRA-S risk groups and epithelial subtypes, or CAPRA-S risk groups and stromal subtypes

	Multivariate cox regression														
	Cohort 1					Cohort 2					Cohort 3				
Variable	N	HR	95% CI	*p* value	C-index	N	HR	95% CI	*p* value	C-index	N	HR	95% CI	*p* value	C-index
CAPRA-S risk group					0.704					0.674					0.738
Low	29	-	-	-		94	-	-	-		69	-	-	-	
Intermediate	60	1.44	0.57–3.64	0.4		142	3.72	1.09–12.7	**0.036**		34	4.49	1.67–12.1	**0.003**	
High	32	5.18	2.07–13.0	**<0.001**		117	7.06	2.13–23.4	**0.001**		19	8.34	2.92–23.8	**<0.001**	
Epithelium															
Subtype 1	60	-	-	-		130	-	-	-		14	-	-	-	
Subtype 3	28	1.61	0.79–3.31	0.2		147	2.25	0.86–3.80	0.12		46	2.04	0.52–7.93	0.3	
CAPRA-S risk group					0.724					0.683					0.765
Low	29	-	-	-		94	-	-	-		69	-	-	-	
Intermediate	60	1.61	0.64–4.08	0.3		142	3.83	0.96–11.6	0.058		34	4.43	1.57–12.0	**0.005**	
High	32	5.43	2.15–13.7	**<0.001**		117	7.95	1.88–22.0	**0.003**		19	6.98	2.43–20.1	**<0.001**	
Stroma															
Subtype 1	43	-	-	-		180	-	-	-		54	-	-	-	
Subtype 3	37	2.67	1.22–5.83	**0.014**		143	2.25	0.86–3.03	0.14		18	2.07	0.73–5.91	0.2	

Significant *p* values (*p* < 0.05) are highlighted.

*HR* hazard ratio, *CI* confidence interval, *C-index* Harrell’s concordance index.

Thus, our data indicate unique transcriptional characteristics inherent to the different epithelial subtypes, potentially underlying distinct PC biology. However, heterogeneity between cohorts in terms of prognostic potential indicates limited clinical utility for subtyping on the epithelial gene expression signature.

### Stromal subtypes are characterized by stromal dysfunction and changes in immune cell infiltration and predict prostate cancer recurrence

**Fig. 3:**
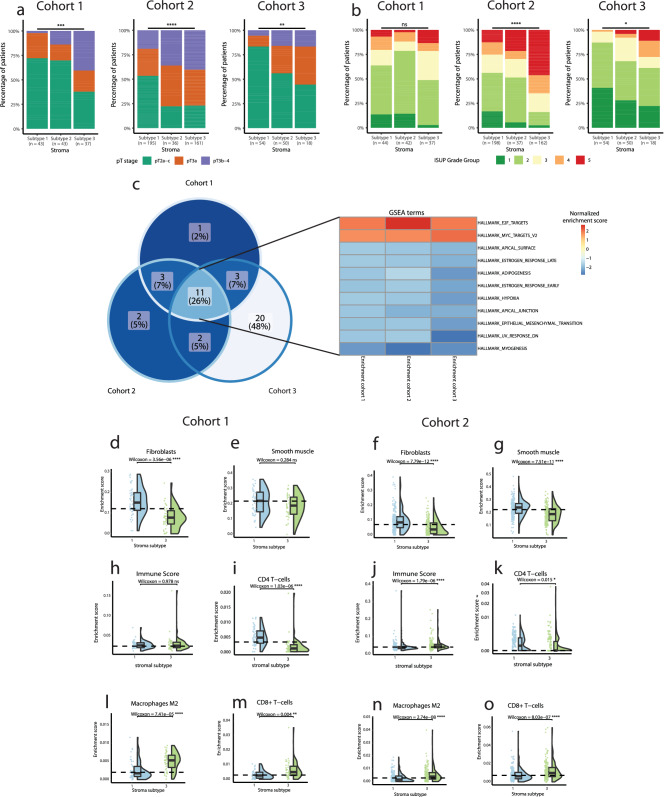
Stromal subtype characteristics. Stacked bar plot in each cohort showing the percentage of patients in each subtype according to (**a**) pathological T-stage, (**b**) 2014 ISUP Gleason grade. Fisher´s Exact test was used to determine significant differences in clinical characteristics. **c** Venn diagram and heatmap showing overlap of significantly enriched Hallmarks for GSEA of DGE between S1 and S3. Raincloud plots show differences in enrichment score between S1 and S3. In cohort 1 for Fibroblasts (**d**), Smooth muscle (**e**), Immune Score (**h**), CD4 + T-cells (**i**), M2-polarized macrophages (**l**), and CD8 + T-cells (**m**). In cohort 2 for Fibroblasts (**f**), Smooth muscle (**g**), Immune Score (**j**), CD4 + T-cells (**k**), M2-polarized macrophages (**l**), and CD8 + T-cells (**m**). Wilcoxon rank-sum test was used to determine significant differences between subtypes S1 and S3. FDR-corrected *p* values are reported. # = axis is square root scaled.

We next sought to characterize and validate the three stromal subtypes, S1, S2 and S3, identified earlier (Fig. 1c). Stromal subtype S3 was associated with higher pT-stage and higher ISUP grade group in all three cohorts, although ISUP grade did not reach statistical significance in cohort 1 (Fig. 3a, b). The subtype with the least (S1) and the subtype with the most (S3) adverse clinicopathological characteristics, respectively, were further compared. Genes from the stroma-specific signature with importance for sample classification into subtypes S1 and S3 have been listed in Supplementary Table 2.

GSEA based on genes differentially expressed between subtype S1 and S3 identified several Hallmark pathways important to PC biology (n = 18, 18, 36 in cohorts 1, 2, and 3, respectively). Of these, 11 pathways were seen to be differentially expressed between S1 and S3 consistently in all 3 cohorts (encompassing 26% of unique enriched pathways across cohorts, *p* < 0.05, Fisher’s Exact Test; Fig. 3c, Supplementary Fig. 6), with alterations such as increases in pathways related to cell growth (E2F and MYC targets), reduced stromal environment functions (e.g., myogenesis, hypoxia, and adipogenesis) and reduced cell polarity (apical junction and apical surface) (Fig. 3c). Thus, indicating that dysregulated stroma is associated with loss of epithelial cell polarity and enhanced tumor growth.

To further investigate this stromal dysregulation, we characterized the cell type compositional differences between S3 and S1 in cohorts 1 and 2 by computational cell type enrichment analysis^26^ (Fig. 3d–o and Supplementary Fig. 7). We observed significantly reduced fibroblast enrichment score in S3 compared to S1 in both cohorts and significantly reduced smooth muscle cell enrichment score in cohort 2 (Fig. 3d–g), corroborating the GSEA findings and indicating that fibroblasts and smooth muscle cells play an important role in maintaining a functional stroma. We did not observe any consistent difference in overall immune cell infiltration between subtype S3 and S1 in cohorts 1 and 2, although, increased immune infiltration was observed in cohort 2 (Fig. 3h, j). In contrast, we observed a significant decrease in CD4 + T-cells in subtype S3 compared to subtype S1 in both cohorts (Fig. 3i, k). Furthermore, a significant increase in M2-polarized macrophages and CD8 + T-cells was observed in subtype S3 in both cohorts (Fig. 3l–o). B-cells, dendritic cells, endothelial cells, eosinophils, epithelial cells, M1 polarized macrophages, mast cells, and regulatory T-cells did not change consistently across the two cohorts (Supplementary Fig. 7). Adipocyte levels were significantly lower in subtype S3, although with many samples in cohort 2 absent for adipocytes (Supplementary Fig. 7).

**Fig. 4:**
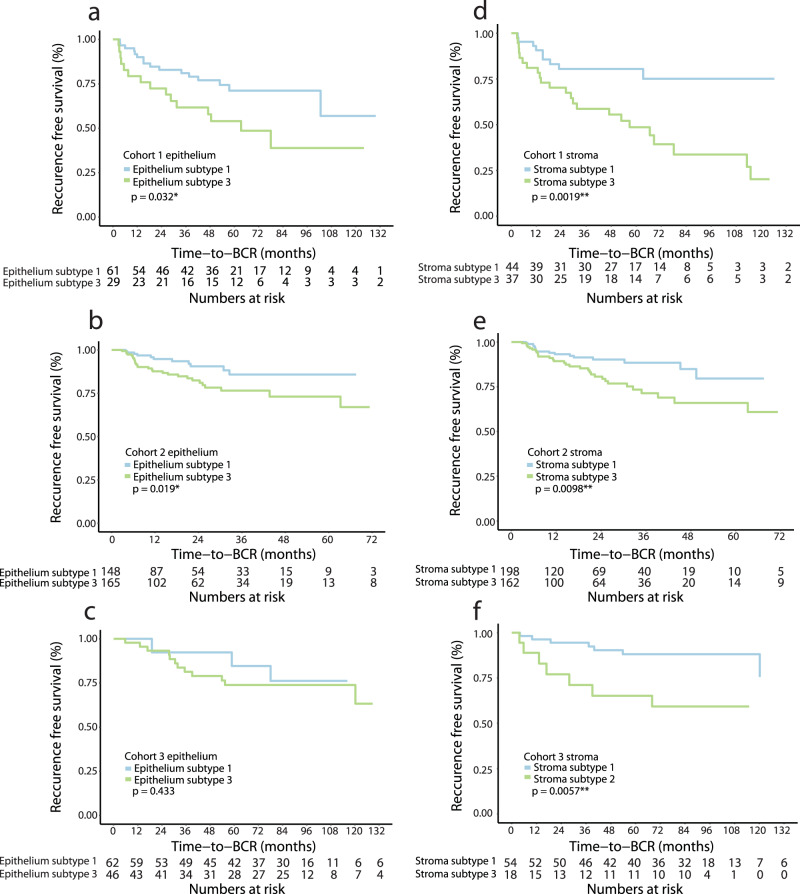
Kaplan-Meier analysis of epithelium and stroma subtypes. Kaplan-Meier plot of BCR risk in E1 and E3 in (**a**) cohort 1, (**b**) cohort 2, and (**c**) cohort 3. Kaplan-Meier plot of BCR risk in S1 and S3 in (**d**) cohort 1, (**e**) cohort 2, and (**f**) cohort 3. Significance determined using log-rank test.

Interestingly, subtype S3 was significantly associated with increased risk of BCR compared to S1 in cohort 1 (log-rank test, *p* = 0.0019, Fig. 4d), cohort 2 (log-rank test, *p* = 0.0098, Fig. 4e), and cohort 3 (log-rank test, *p* = 0.0057, Fig. 4f). These results were corroborated by univariate Cox regression analyses of BCR-free survival in subtype S3 compared to S1 (Hazard Ratio (HR) = 3.2, HR = 2.14, HR = 3.42 in cohort 1, 2, and 3, respectively, *p* < 0.05; Table 2). In multivariate analyses, subtype S3 was significantly associated with BCR independent of CAPRA-S risk group in cohort 1, but not in cohorts 2 and 3 (Table 3).

To validate that the observed differences in recurrence risk between stromal subtypes was associated to stroma-specific gene expression and not to unexplored background processes inherent to the datasets, we stratified patients on the 500 most variably expressed genes in cohort 1 (Supplementary Fig. 1). Association of BCR risk with patient subgroups stratified on variably expressed genes would indicate that unexplored biological processes in the tissue could be responsible for the trends in the dataset attributed to stromal characteristics. Stratification of patients on the 500 most variably expressed genes revealed stable clusters in all three cohorts, but were not significantly associated with prognostic outcome (BCR) (Supplementary Fig. 8). Together, this indicates that although some background processes may be present in the dataset due to the stable clustering, these could not replicate the prognostic potential observed from stromal subtypes. Hence, the subtypes based on stroma specific gene expression could not be attributed to underlying background gene expression.

Taken together, our results indicate that stromal transcriptional dysregulation and cell type changes inherent to the stromal subtypes underlie distinct PC biology and disease aggressiveness. This was corroborated by the consistent prognostic potential of these subtypes across three large independent RP cohorts from multiple countries.

### Stromal subtypes improve risk stratification of prostate cancer patients with intermediate risk

To further improve on the prognostic effect of the subtypes, we investigated prognostic ability of the overlap between high-risk epithelial and stromal subtypes, as well as between low-risk epithelial and stromal subtypes (Supplementary Fig. 9a-c). However, stratification of patients into combined high-risk (S3 + E3, S3 + E2, S2 + E3) or combined low-risk (S1 + E1, S1 + E2, S2 + E1) did not robustly increase prognostic accuracy compared to S3 alone (Fig. 4d–f compared to Supplementary Fig. 9a-c).

**Fig. 5:**
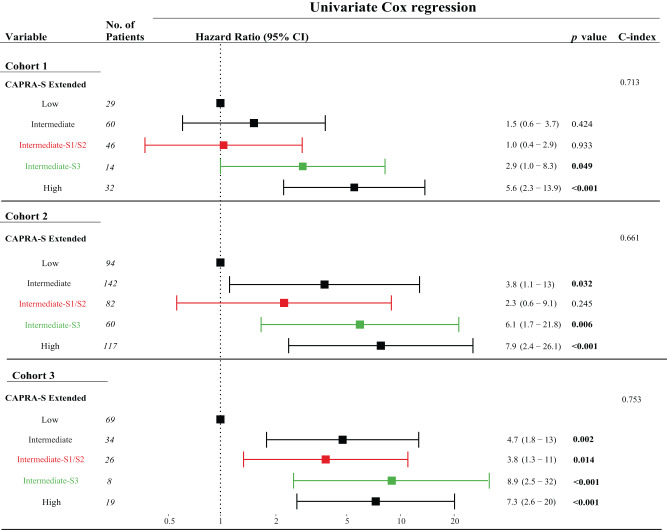
Forest plots of univariate Cox regression analysis using stromal subtypes to re-stratify CAPRA-S intermediate risk. Significant *p* values (*p* < 0.05) are highlighted.

Instead, since we identified stromal subtypes to harbor robust prognostic potential, we asked if this stroma-derived subtype information could be used to further improve upon the current prognostic risk evaluation tools. To test this, we selected patients with an ambiguous, intermediate risk of BCR according to CAPRA-S. Interestingly, CAPRA-S intermediate risk patients with the S3 subtype had an increased BCR risk compared to CAPRA-S intermediate-risk patients with S1/S2 subtypes in cohort 1 (log-rank test, Supplementary Fig. 10a, *p* = 0.032). This was validated in cohort 2 and cohort 3 (Supplementary Fig. 10b, c, Supplementary Table 3) and could not be recreated using the epithelial subtypes instead (CAPRA-S intermediate + E1/E2 vs. CAPRA-S intermediate + E3) (Supplementary Fig. 10d-f, Supplementary Table 3). Additionally, univariate Cox regression showed the conjugated CAPRA-S intermediate + S3 subgroup to have BCR risk comparable to that of the CAPRA-S high risk group (cohort 1, delta HR = 1.4, Fig. 5). This re-stratification of the BCR risk in the CAPRA-S intermediate + S3 subgroup was further validated in cohort 2 and cohort 3 (cohort 2, delta HR = 2.3; cohort 3, delta HR = 4.2; Fig. 5, Supplementary Table 3).

Thus, our results suggest a potential for further stratification of clinically defined (CAPRA-S) intermediate risk PC based on stroma-derived molecular risk classification. Taken together our results indicate that, beyond cancer cell (epithelial) characteristics, the surrounding stromal environment holds unique biological characteristics that can be exploited for prognostication in conjunction with clinical variables.

## Discussion

There is mounting evidence that characterization of the TME, with a focus on both immune cell and stromal characteristics, can increase our understanding of PC pathobiology and be used to improve prognostic accuracy^7,24^. To this end, we clustered LPC patients into subtypes based specifically on epithelial or stromal gene expression. The emerging subtypes showed unique characteristics for both the stromal and epithelial subtypes, however only the stromal subtypes showed consistent prognostic potential across the training cohort and two external validation cohorts.

Characterization of the three stromal subtypes showed subtype S3 to be associated with adverse clinical changes and subtype S1 to be associated with more indolent characteristics (Fig. 3a, b), thus indicating that stroma-associated gene expression could be biologically relevant for determining PC aggressiveness. While previous studies have used gross stromal histology or the presence of individual cell types as markers for PC aggressiveness^27,28^, these have seen limited clinical relevance and consequently have not been translated into clinical practice. Interestingly, in our study, although subtype identification was without any information on malignancy, the stromal subtypes displayed clear and robust clinical and prognostic differences in multiple independent cohorts (Fig. 3a, b, Fig. 4d-f). To the best of our knowledge, the current study is the first to use unsupervised clustering to identify clinically relevant TME subtypes in PC and suggests that the stromal subtypes identified in this study could hold prognostic potential in clinically ambiguous PC, although evaluation of prognostic potential requires further studies.

Multiple transcriptional pathways and cell types differed between the stromal subtypes S1 and S3. Of these, the decreased fibroblast and smooth muscle cell enrichment in subtype S3 (Fig. 3d-g) likely reflects a larger change in the stromal composition, as also corroborated by the observed transcriptional changes in subtype S3 (e.g., myogenesis, adipogenesis, and hypoxia, Fig. 3c). Beyond the stromal changes, subtype S3 also had reduced infiltration of CD4 + T-cells, which have been associated to reduced risk of PC lymph node metastasis^29^. Furthermore, subtype S3 had increased infiltration of M2-polarized macrophages, which have previously been associated to metastatic potential and interactions with cancer associated fibroblasts in PC^15,30^, and increased CD8 + T-cells, where no consensus on influence on PC prognosis have been reached^16,27,31,32^. Thus, increased levels of M2 polarized macrophages likely reflects an immunosuppressive environment in subtype S3^33^, while change in the specific T-cell subsets require additional investigation. Interestingly, the changes in stromal and immune cell composition and failure to maintain stromal functions were correlated to increased cell growth and a loss of epithelial cell polarity, processes that are invariably linked to cancer progression and metastatic spread^34,35^. This suggests a role of stromal and immune cells in the dysregulation to the stromal environment that may be necessary for the transformation of LPC into metastatic PC.

While the stromal subtypes predicted disease aggressiveness across multiple cohorts (Fig. 4d-f), these results could not be replicated using the epithelial subtypes (Supplementary Fig. 10d-f) nor substantially improved by addition of epithelial subtype information to stromal subtypes (Supplementary Fig. 9a-c, compared to Fig. 4d-f). Furthermore, by adding the stromal subtype information to the CAPRA-S risk groups we saw increased prognostic ability (Fig. 5), highlighting the possible utilization of integrating stromal information for patients where tumor-centric information (such as Gleason grading, T-stage, and serum PSA level) is inadequate to provide a clear outcome prediction.

Indeed, Gleason grade group and T-stage evaluation may already explain much of the malignant transformation in PC epithelial cells that genomic subtypes in LPC attempt to identify^36,37^. While the molecular heterogeneity of primary PC is well documented^38^, TME heterogeneity in PC is largely unexplored. Thus, the stromal changes we identify across cohorts may reflect more universal changes to the TME in aggressive cancer, beyond heterogeneous epithelial (malignant cell) characteristics. Our results support the hypothesis that although cancer cells have the capacity to disseminate and become metastatic, a feature that can somewhat be discerned from the histology (i.e., high Gleason Grade), an altered stromal environment is also required for further evolution of the cancer and metastatic spread^19,39^.

Pending further validation, the link between stromal composition and clinical outcome could potentially be utilized in a clinical setting in the future. Risk stratification and management of intermediate-risk PC with active surveillance is controversial and disputed as a viable treatment strategy^40^. Here, utilization of stromal subtyping may aid in improving risk stratification and tracking of metastatic potential. In line with this, offering active treatment to intermediate-risk patients with stromal high-risk disease may then improve the safety of referring the remaining intermediate-risk PC patients to AS. Furthermore, commercial gene expression tests available for PC treatment guidance have been investigated for use during active surveillance of intermediate-risk PC, but were not able to predict disease upstaging^41^. Inclusion of stromal subtype evaluation in the continuous surveillance of intermediate-risk PC patients may also have the potential to improve prediction of progression risk. However, clinical studies utilizing stromal subtyping for treatment selection or continuous evaluation of progression risk is needed to prove any such potential utility.

Although several commercial and non-commercial gene expression-based models are available for prostate cancer treatment guidance, only a few utilize stromal gene expression^42–44^. While most models rely on expression of cell-cycle or growth related genes, the Oncotype DX model includes multiple genes related to stromal composition and cellular organization^45^. Several of the Oncotype DX genes were present either in our stromal signature (*COL1A1*, *FLNC, GSN* and *TPM2*) or epithelial signature (*KLK2* and *AZGP1*), with stromal signature genes being related to stromal response and cellular organization^45^. Likely, the stromal genes in Oncotype DX quantify some of the aspects captured by our stromal subtypes. Hence, other stromal subtype characteristics could further improve this model, and other prognostic models, especially for evaluation of intermediate risk PC. Future studies should investigate this further.

In addition to the gene-expression-based models, other studies have developed stroma-related PC signatures for prediction of high Gleason PC or metastatic potential following RP^19,39^. One study used LCM samples of epithelium and stroma from patients with low (3 + 3) or high (≥8) Gleason score PC to develop a 24-gene signature, which they named the Gleason stromal gene signature. Interestingly, where we observed limited prognostic utility between epithelial subtypes in the current study, this previous study identified only one epithelial gene with consistent expression change between low and high Gleason grade PC, corroborating the difficulty of identifying prognostic epithelial subtypes in PC. While the Gleason stromal gene signature was able to separate low and high Gleason grade PC, including in a recent validation study^46^, no further evaluation of its clinical utility beyond Gleason grade prediction has been performed.

Another study used a pre-clinical, patient-derived xenograft model of PC metastasis in mice to develop a 93-gene stroma-derived metastatic signature (SDMS)^19^. The SDMS stratified patients into high or low risk of metastasis following RP. Indeed, similar to how our S3 subtype improved risk stratification for patients with CAPRA-S intermediate risk PC (Fig. 5), the SDMS improved risk stratification for patients with Gleason score seven tumors. While the prognostic value of the SDMS was validated in multiple independent cohorts, the study used a murine model lacking several immune components for signature development potentially limiting insight into the stroma and immune cell interplay in PC progression. A total of 5 genes (*AEBP1, C1S, COL1A1, LUM*, *PRELP*) overlap between our stromal signature and the Gleason stromal gene signature by Tyekucheva et al ^19^., while seven genes (*ACTG2, DES, MGP, SPARC, TPM1, CLU, COL14A1)* overlap between our stromal signature and the SDMS signature reported by Mo et al. ^39^. The small overlap in genes between our stromal signature and both the previously reported Gleason stromal gene signature^19^ and SDMS^39^ highlight that both the methods used to identify the different stromal signatures, and the underlying biological differences identified, are different between the three studies. The previously identified signatures were based on specific clinical differences (i.e., low vs. high Gleason, metastatic vs. non-metastatic), while our stromal signature was based on benign prostate tissue, but turned out to be clinically relevant, likely due to identified differences in microenvironment composition and function. Nonetheless, the biological aspects of PC progression identified with the Gleason stromal gene signature, the SDMS, and our stromal subtypes all provide unique insights into a more holistic understanding of the PC TME and its role in malignancy.

Our study has certain limitations. First, the use of BCR as endpoint for prognostic evaluation instead of the more clinically appropriate metastatic spread or PC-specific death. However, as early-stage PC is characterized by a long time to progression^47^, it requires more than 10 years of follow-up to evaluate these outcomes. Secondly, no spatial or single cell data was available to perform more in-depth analysis of the subtypes. Instead, our study utilized several large, independent, and international cohorts of bulk tumor transcriptomic data to identify subtypes using unsupervised clustering, and to evaluate subtype aggressiveness using follow-up information. Single cell or high-resolution spatial transcriptomic dataset with fewer samples and short follow-up would have been inadequate for such analyses. Further work should investigate details of the now established stromal subtypes using single cell or high-resolution spatial transcriptomic analysis of PC tissue.

In conclusion, we have identified stromal subtypes in primary tumor samples of LPC patients and validated their characteristics in two independent cohorts. We have identified a stromal subtype (S3) that was consistently more aggressive in LPC, displayed specific stromal dysfunction at both the cellular and pathways level, and had increased M2-polarized macrophage and CD8 + T-cell infiltration. Furthermore, it improved prognostic stratification of patients with clinically intermediate risk PC, which suggest potential use in active surveillance management of intermediate risk disease. However, further studies validating the clinical utility of stromal subtypes in PC is required.

## Methods

### **Patient cohorts**

Cohort 1 included 127 fresh-frozen primary PC tissue specimens and 31 AN prostate specimens from 142 patients with LPC treated by RP (Table 1). Samples were collected at the Department of Urology, Aarhus University Hospital (2004–2017) or Regional Hospital West Jutland (2016–2019). RNA profiling was performed by total RNA-sequencing as previously described^48,49^.

All research for cohort 1 was carried out in accordance with the principles of the Helsinki Declaration and was approved by The Central Denmark Region Committees on Health Research Ethics [#2000/0299, #1–10–72–361–18, #1–10–72–367–13], The National Committee on Health Research Ethics [#1603543/66451] and notified to The Danish Data Protection Agency [#2013–4–2041, #1–16–02–330–13, #1–16–02–23–19, #1–16–02–248–14]. Written consent was obtained from all participants prior to their donation of tissue samples for a research biobank, while the requirement for patient consent to the specific analyses in this retrospective study was waived.

Cohort 2 included 406 tumor samples from patients with LPC treated by RP from The Cancer Genome Atlas Prostate Adenocarcinoma Dataset (TCGA-PRAD)^36^. RNA profiling of tumor samples was performed by poly(A) enriched RNA sequencing. Molecular and clinical data was publicly available and obtained from the TCGA data portal^50^ as described previously^51^.

Cohort 3 included 126 tumor samples from patients with LPC treated by RP from the Memorial Sloan-Kettering Cancer Center dataset by Taylor et al. (MSKCC)^52^. RNA profiling of tumor samples was conducted using an Affymetrix Human Exon 1.0 ST Array. Expression data was publicly available and obtained from the Gene Expression Omnibus (GEO) database (GSE21032).

The laser-capture microdissection dataset included 5 cystoprostatectomy samples from patients with bladder cancer from Tyekucheva et al. ^19^. Samples were reviewed for no incidental prostate cancer by a pathologist and laser capture microdissection was used to isolate areas of epithelium or stroma^19^. RNA profiling was performed using the Affymetrix Gene Array STA 1.0. Expression data was publicly available and obtained from the GEO database (GSE97284).

### **RNA extraction and sequencing**

For cohort 1, total RNA extraction and library preparation was performed as previously described^24,49^. Briefly, immediately following RP, fresh prostate tissue biopsy samples were obtained and stored at −80 °C in TissueTek. Approximately 40 sections (20 µm thick) were cut from each sample. The first and last tissue sections were stained with Hematoxylin and Eosin (H&E) and evaluated by a pathologist for areas of malignant prostate tissue or benign prostate tissue absent of malignant cells. Total RNA extraction was performed on the remaining sections using the RNeasy Plus Mini Kit (QIAGEN, Cat#74036). RNA concentration was assessed on a NanoQuant Plate™ (TECAN). RNA quality was assessed using a 2100 Bioanalyzer (Agilent).

Sequencing libraries were generated using the ScriptSeq RNA-seq Library kit with RiboZero™ Magnetic Gold Kit (Illumina; AN = 11, LPC = 52) or the KAPA RNA HyperPrep Kit with KAPA RiboErase Kit (Roche; AN = 20, LPC = 75). Paired-end sequencing was performed using either Illumina HiSeq 2000, NextSeq 500, or NovaSeq 6000 ( ~ 25 million reads/sample; 2 × 75 bp, 2 × 75 bp, or 2 ×100 bp, respectively). All reads were QC checked and trimmed, and transcripts were quantified using Kallisto (version 0.46.2)^53^ with GrCh38.p13/hg38 as reference transcriptome. Transcripts were summarized to gene level counts using tximport^54^. Subsequently, normalization, filtering and log2 transformation was performed using edgeR^55^. Correction for batch effects was performed in the design formula for differential expression analyses or using the removeBatchEffect function from the R package Limma^56^.

### Gene signature definition and subtype identification

Genes for the epithelium- and stroma-specific signatures were identified in an external laser-capture microdissection dataset (see above), based on two criteria: 1) differential gene expression analysis of epithelium vs. stroma using Limma^56^, where we selected genes significantly upregulated in either healthy prostate epithelium or healthy prostate stroma (false discovery rate (FDR) < 0.05, log2 fold-change (logFC) ≥ 2.0). And 2) Gene Ontology^57,58^ analysis for biological processes, where the final selection required significant enrichment of epithelium- or stroma-specific Gene Ontology terms (Fold enrichment ≥10, FDR < 0.05) when using epithelium or stroma genes, respectively, for the analysis. For the epithelium genes, one pathway was significantly enriched: Epithelial cell development. For the stromal genes, seven pathways were significantly enriched and all related to stromal functions: Regulation of complement activation, Regulation of transforming growth factor-beta secretion, Mesenchymal migration, Regulation of amyloid fibril formation, Regulation of smooth muscle cell migration, and Regulation of metallopeptidase activity. This resulted in 86 epithelium-associated genes for an epithelial signature and 88 stroma-associated genes for a stromal signature (Supplementary Table 1).

For the epithelial subtype and the stromal subtype discovery in cohort 1, patient clustering was performed on the epithelial signature (n = 86 genes) or the stromal signature (n = 88 genes), respectively, using NMF with consensus clustering via the R package NMF^59^. The optimal number of clusters/subtypes for each signature was evaluated based on the cophenetic and silhouette scores, indicative of clustering stability. Based on an initial evaluation of a range of clusters (2–8 clusters), having three clusters was deemed as being optimal both when clustering based on the epithelial and stromal signature and hence used for the final consensus clustering with 5000 clustering iterations. Genes important for sample placement into stromal subtype S1 or S3 were extracted using the feature selection method described in Carmona-Saez et al. ^60^. implemented in the NMF package in R (Supplementary Table 2).

### **Validation of epithelial and stromal subtypes**

Subtype validation in cohort 2 and cohort 3 was performed on samples clustered on identical gene-expression signatures and clustering parameters to cohort 1 (n = 3 clusters, iterations = 5000). Cluster similarity across cohorts was validated using Spearman’s rank correlation. Spearman’s rank correlation analysis was used to evaluate the concordance of gene contribution to placement of samples into a specific subtype (gene weights) across cohorts. A positive correlation of gene weights confirmed that E1, E2 and E3 were defined based on the same epithelial genes across the three cohorts (Supplementary Fig. 4a). Similarly, correlation of gene weights validated that subtypes S1 and S3 were based on the same stromal genes across the three cohorts, while S2 could not be validated in all three cohorts (Supplementary Fig. 4b).

### Differential gene expression analysis and gene set enrichment analysis

Transcriptomic differences between AN and LPC samples in cohort 1 were examined by DGE analysis using edgeR^55^. Transcriptomic differences between the high- and low-risk stromal subtypes (S3 and S1) and between the high- and low-risk epithelial subtypes (E3 and E1) were examined by DGE analysis. In cohorts 1 and 2 (RNA sequencing data) DGE was performed using edgeR, while in cohort 3 (microarray expression data) DGE was performed using Limma in R^56^. In all cases, adjustment for multiple testing was performed using the Benjamini-Hochberg (FDR) method^61^. Adjusted *p* values < 0.05 were considered significant.

Pre-ranked GSEA was subsequently performed on the output from either edgeR or Limma. GSEA was performed using the R package fgsea^62^ based on the cancer Hallmark Gene Set Collection from the Molecular Signature Database^63^. Gene sets with BH-adjusted *p* value < 0.05 were considered significantly enriched, while normalized enrichment scores were used to determine the level of enrichment.

### Cell-type enrichment analysis

The cellular composition of the subtypes was estimated by cell type enrichment analysis using the xCell^26^ package for R. xCell uses bulk gene expression data to determine the enrichment of specific cell types in individual samples, based on cell type-specific expression signatures. Cell type enrichment analysis was evaluated in the cohorts with tumor RNA sequencing data (cohort 1 and cohort 2), as the microarray data format available from cohort 3 was not available to us in a format eligible for accurate xCell analysis^64^.

### **Statistical analysis**

All statistical analyses were performed in R (v. 4.0.2) using R Studio (v. 1.1.463). Comparison of categorical variables (i.e., clinicopathological parameters) was conducted using Fisher’s exact Test, while comparison of continuous variables (e.g., cell type enrichment) was done using Wilcoxon rank-sum test with BH-corrected *p* values.

For comparison of stromal and epithelial subtypes to clinicopathologically defined risk groups, we used the established CAPRA-S nomogram^3^. CAPRA-S risk groups were defined based on scores [0–2] = low risk; [3–5] = intermediate risk; [6–12] = high risk^3^. Stromal subtypes, epithelial subtypes, and CAPRA-S risk groups were analyzed as categorical variables in survival analyses. The prognostic potential of the subtypes and CAPRA-S risk groups was evaluated by BCR-free survival analyses, using univariate and multivariate Cox regression, Kaplan-Meier and log-rank tests in the survival package in R^65^. BCR was defined as PSA ≥ 0.2 ng/mL. Patients without BCR were censored at their most recent PSA measurement. Patients with unknown BCR status (lost to follow-up) or BCR within 3 months of RP (likely to have residual tumor) were excluded from BCR-free survival analyses (cohort 1, *n* = 3; cohort 2, *n* = 9; cohort 3, *n* = 4). Prognostic accuracy was determined using Harrell’s C-index.

### Reporting summary

Further information on research design is available in the Nature Research Reporting Summary linked to this article.

### Supplementary information

REPORTING SUMMARY

Supplementary Materials

## Data Availability

As the requirement for patient consent was waived in the current study, we do not have permission to deposit the full raw and individual-level clinical data in a public repository. The raw data, individual-level clinical data, and processed gene expression files for the discovery cohort have instead been deposited in the controlled access repository the GenomeDK Data Library (ID GDK000002). All inquiries regarding data access should be made to the Data Access Committee as described on the project’s GenomeDK Data Library page (https://genome.au.dk/library/GDK000002/).

## Code availability

The underlying code for this study is available from the corresponding author upon reasonable request.
