# Supplementary material for: Stroma-specific gene expression signature identifies prostate cancer subtype with high recurrence risk
Source: NPJ Precis Oncol. 2024 Feb 23;8:48. doi: 10.1038/s41698-024-00540-x (PMC10891092; doi:10.1038/s41698-024-00540-x)
Supplement: Supplementary file 1 — REPORTING SUMMARY [file 41698_2024_540_MOESM1_ESM.pdf]

nature portfolio

Corresponding author(s): KARINA DALSGAARD SØRENSEN

Last updated by author(s): Nov 29, 2023

Reporting Summary

Nature Portfolio wishes to improve the reproducibility of the work that we publish. This form provides structure for consistency and transparency in reporting. For further information on Nature Portfolio policies, see our Editorial Policies and the Editorial Policy Checklist.

Statistics

For all statistical analyses, confirm that the following items are present in the figure legend, table legend, main text, or Methods section.

n/a	Confirmed	
☐	☒	The exact sample size (*n*) for each experimental group/condition, given as a discrete number and unit of measurement
☐	☒	A statement on whether measurements were taken from distinct samples or whether the same sample was measured repeatedly
☐	☒	The statistical test(s) used AND whether they are one- or two-sided<br>*Only common tests should be described solely by name; describe more complex techniques in the Methods section.*
☐	☒	A description of all covariates tested
☐	☒	A description of any assumptions or corrections, such as tests of normality and adjustment for multiple comparisons
☐	☒	A full description of the statistical parameters including central tendency (e.g. means) or other basic estimates (e.g. regression coefficient) AND variation (e.g. standard deviation) or associated estimates of uncertainty (e.g. confidence intervals)
☐	☒	For null hypothesis testing, the test statistic (e.g. *F*, *t*, *r*) with confidence intervals, effect sizes, degrees of freedom and *P* value noted<br>*Give P values as exact values whenever suitable.*
☒	☐	For Bayesian analysis, information on the choice of priors and Markov chain Monte Carlo settings
☒	☐	For hierarchical and complex designs, identification of the appropriate level for tests and full reporting of outcomes
☒	☐	Estimates of effect sizes (e.g. Cohen's *d*, Pearson's *r*), indicating how they were calculated

Our web collection on statistics for biologists contains articles on many of the points above.

Software and code

Policy information about availability of computer code

Data collection	Kallisto (version 0.46.2) with GrCh38.p13/hg38 as reference transcriptome was used to quantify data. Additional data was obtained from publicly avaiable datasets.
Data analysis	All statistical analyses were performed in R (v. 4.0.2) using R Studio (v. 1.1.463). The underlying code for this study is available from the corresponding author.

For manuscripts utilizing custom algorithms or software that are central to the research but not yet described in published literature, software must be made available to editors and reviewers. We strongly encourage code deposition in a community repository (e.g. GitHub). See the Nature Portfolio guidelines for submitting code & software for further information.

Data

Policy information about availability of data

All manuscripts must include a data availability statement. This statement should provide the following information, where applicable:

- Accession codes, unique identifiers, or web links for publicly available datasets
- A description of any restrictions on data availability
- For clinical datasets or third party data, please ensure that the statement adheres to our policy

As the requirement for patient consent was waived in the current study, we do not have permission to deposit the full raw and individual level clinical data in a public repository. The raw data, individual level clinical data, and processed gene expression files for the discovery cohort have instead been deposited in the

controlled access repository the GenomeDK Data Library (ID GDK000002). All inquiries regarding data access should be made to the Data Access Committee as described on the project's GenomeDK Data Library page (https://genome.au.dk/library/gdk000002/).

Research involving human participants, their data, or biological material

Policy information about studies with human participants or human data. See also policy information about sex, gender (identity/presentation), and sexual orientation and race, ethnicity and racism.

Reporting on sex and gender	Prostate cancer only affect patients of male sex. No analysis of or reporting on sex or gender was performed in this study.
Reporting on race, ethnicity, or other socially relevant groupings	No reporting on race, ethnicity or, other socially relevant groupings, was performed in this study.
Population characteristics	Population characteristics are summarized in Table 1.
Recruitment	Samples were collected from patients undergoing Radical Prostatectomy at the Department of Urology, Aarhus University Hospital (2004-2017) or Regional Hospital West Jutland (2016-2019). Written consent was obtained from all participants prior to their donation of tissue samples for a research biobank.
Ethics oversight	Ethics, consent and permission.<br>All research was carried out in accordance with the principles of the Helsinki Declaration and was approved by The Central Denmark Region Committees on Health Research Ethics [#2000/0299, #1-10-72-361-18, #1-10-72-367-13], The National Committee on Health Research Ethics [#1603543/66451] and notified to The Danish Data Protection Agency [#2013–41-2041, #1-16-02-330-13, #1-16-02-23-19, #1-16-02-248-14]. Written consent was obtained from all participants prior to their donation of tissue samples for a research biobank, while the requirement for patient consent to the specific analyses in this retrospective study was waived.

Note that full information on the approval of the study protocol must also be provided in the manuscript.

Field-specific reporting

Please select the one below that is the best fit for your research. If you are not sure, read the appropriate sections before making your selection.

☒ Life sciences ☐ Behavioural & social sciences ☐ Ecological, evolutionary & environmental sciences

For a reference copy of the document with all sections, see nature.com/documents/nr-reporting-summary-flat.pdf

Life sciences study design

All studies must disclose on these points even when the disclosure is negative.

Sample size	No sample size calculation was conducted for this study. Discovery cohort size was based on number of samples avaiable for total RNA sequencing. Publicly avaiable datasets were downloaded and analyzed in their full sizes.
Data exclusions	No data was systematically excluded from this study.
Replication	Bioinformatic findings were validated in two independent publicly available cohorts. No experimental data was attempted validated in this study.
Randomization	Sample groups were defiend based on biological phenotypes. No randomization was applied.
Blinding	Blinding not applicable

Reporting for specific materials, systems and methods

We require information from authors about some types of materials, experimental systems and methods used in many studies. Here, indicate whether each material, system or method listed is relevant to your study. If you are not sure if a list item applies to your research, read the appropriate section before selecting a response.

Materials & experimental systems

n/a	Involved in the study
☒	☐ Antibodies
☒	☐ Eukaryotic cell lines
☒	☐ Palaeontology and archaeology
☒	☐ Animals and other organisms
☒	☐ Clinical data
☒	☐ Dual use research of concern
☒	☐ Plants

Methods

n/a	Involved in the study
☒	☐ ChIP-seq
☒	☐ Flow cytometry
☒	☐ MRI-based neuroimaging

Plants

Seed stocks	*Report on the source of all seed stocks or other plant material used. If applicable, state the seed stock centre and catalogue number. If plant specimens were collected from the field, describe the collection location, date and sampling procedures.*
Novel plant genotypes	*Describe the methods by which all novel plant genotypes were produced. This includes those generated by transgenic approaches, gene editing, chemical/radiation-based mutagenesis and hybridization. For transgenic lines, describe the transformation method, the number of independent lines analyzed and the generation upon which experiments were performed. For gene-edited lines, describe the editor used, the endogenous sequence targeted for editing, the targeting guide RNA sequence (if applicable) and how the editor was applied.*
Authentication	*Describe any authentication procedures for each seed stock used or novel genotype generated. Describe any experiments used to assess the effect of a mutation and, where applicable, how potential secondary effects (e.g. second site T-DNA insertions, mosiacism, off-target gene editing) were examined.*
