# Supplementary material for: Stroma-specific gene expression signature identifies prostate cancer subtype with high recurrence risk
Source: NPJ Precis Oncol. 2024 Feb 23;8:48. doi: 10.1038/s41698-024-00540-x (PMC10891092; doi:10.1038/s41698-024-00540-x)
Supplement: Supplementary file 2 — Supplementary Materials [file 41698_2024_540_MOESM2_ESM.pdf]

Supplementary Figure 1

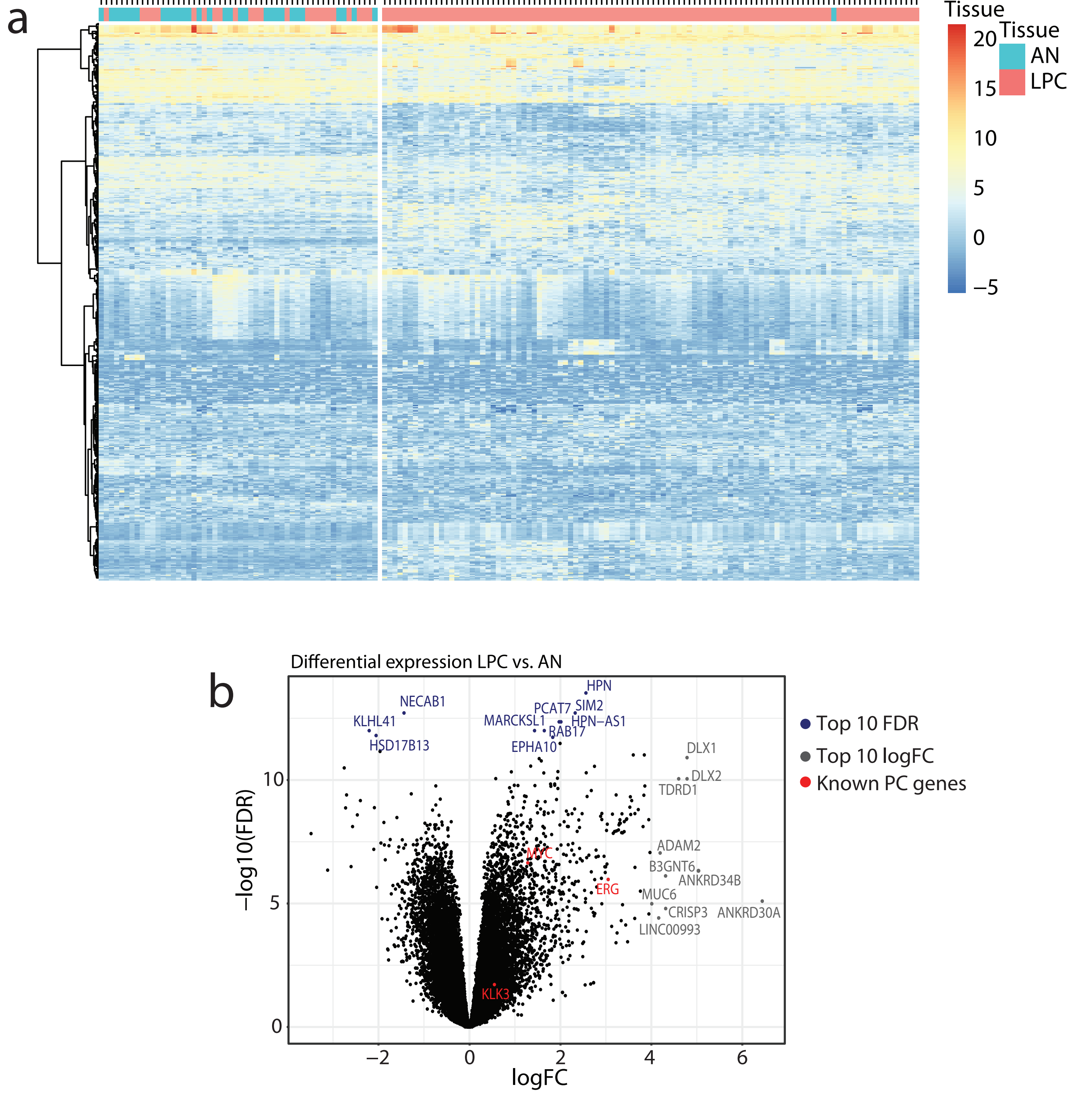

Supplementary Figure 1. Clustering and Differential expression profiling of prostate cancer RNA sequencing cohort 1. (**a**) Heatmap on expression and hieratical clustering of the 500 most variable genes all samples. Samples are labelled by their tissue of origin and coloured on gene expression in counts per million reads. (**b**) Volcano plot showing differentially expressed genes in the comparison between localized prostate cancer and adjacent normal samples. Genes are labelled in the plot if they are among the top 10 most significant deregulated genes (FDR), the top 10 most overexpressed genes (logFC), or the three known PC related genes *ERG*, *MYC* and *KLK3*. *FDR* Benjamini-Hochberg adjusted *p* value, *logFC* log2 fold change.

Supplementary Figure 2

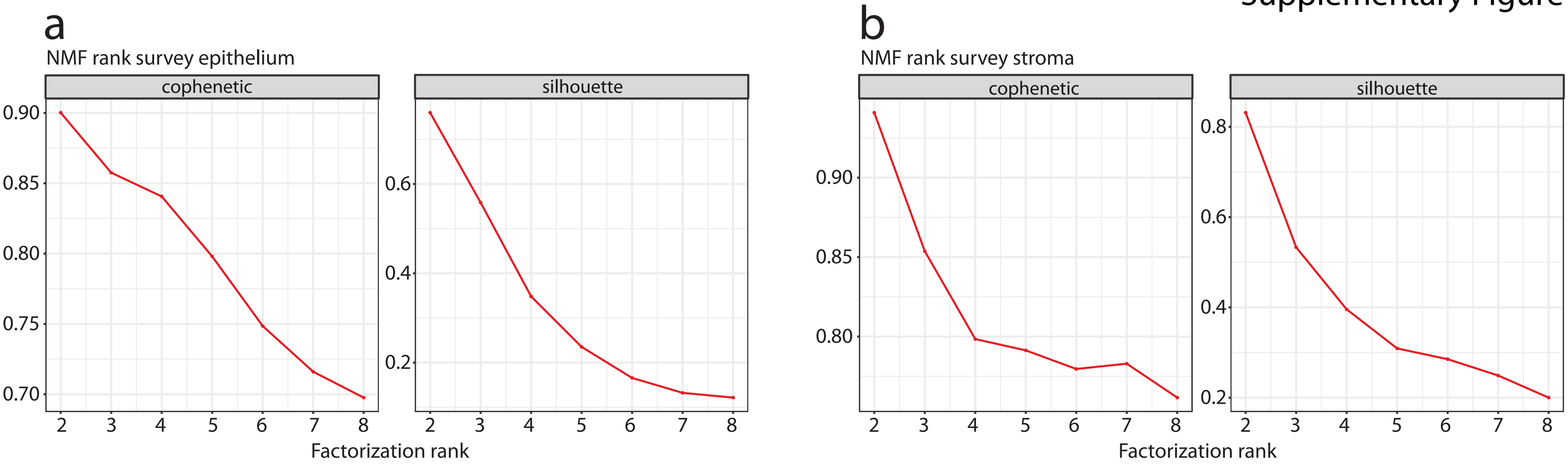

Supplementary Figure 2. Characteristics of NMF consensus clustering on specific gene-signatures. Cophenetic and silhouette scores of NMF consensus clustering into 2-8 groups based on (**a**) epithelium specific gene-expression signature or (**b**) stroma specific gene-expression signature.

Supplementary Figure 3

a

	Stroma S1	Stroma S2	Stroma S3
Epithelium E1	22.8%	24.4%	1.6%
Epithelium E2	7.1%	8.7%	11.8%
Epithelium E3	4.7%	1.6%	17.3%

b

	Stroma S1	Stroma S2	Stroma S3
Epithelium E1	26.1%	2.7%	8.1%
Epithelium E2	9.1%	2.5%	10.3%
Epithelium E3	14.3%	4.2%	22.7%

c

	Stroma S1	Stroma S2	Stroma S3
Epithelium E1	27.0%	18.3%	4.0%
Epithelium E2	4.0%	7.1%	0.8%
Epithelium E3	12.7%	14.3%	11.9%

Supplementary Figure 3. Overlap in epithelial and stromal subtypes. Patient overlap between the three Epithelial and three Stromal subtypes in cohort 1 (**a**), cohort 2 (**b**) and cohort 3 (**c**).

Supplementary Figure 4

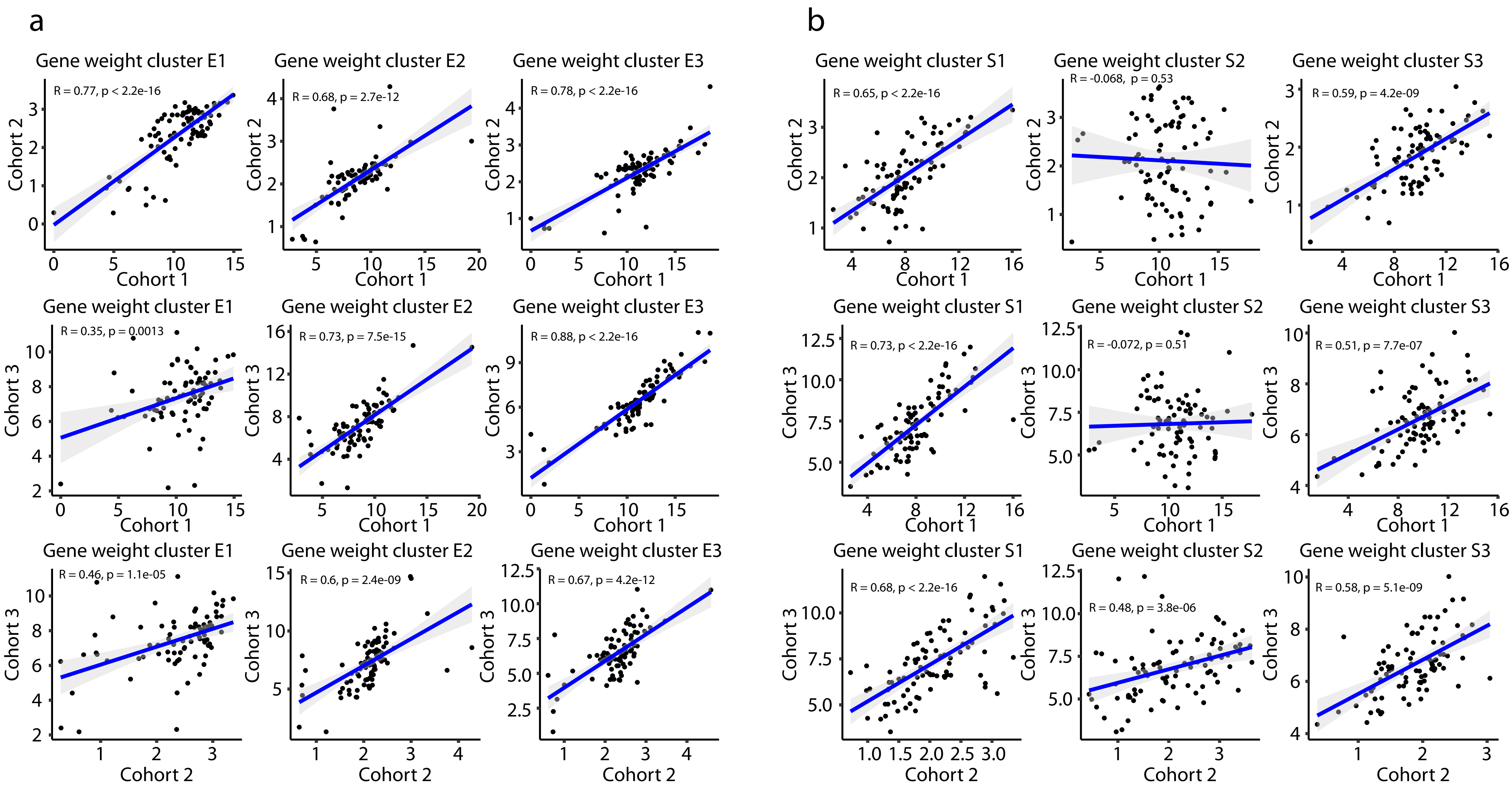

Supplementary Figure 4. Correlation of gene weight in NMF consensus clustering. Correlation of gene weight for placement into each subtype across the three cohorts for (**a**) the epithelium gene-expression signature and (**b**) the stromal gene-expression signature. Correlation and significance based on spearman's rank correlation coefficient.

Supplementary Figure 5

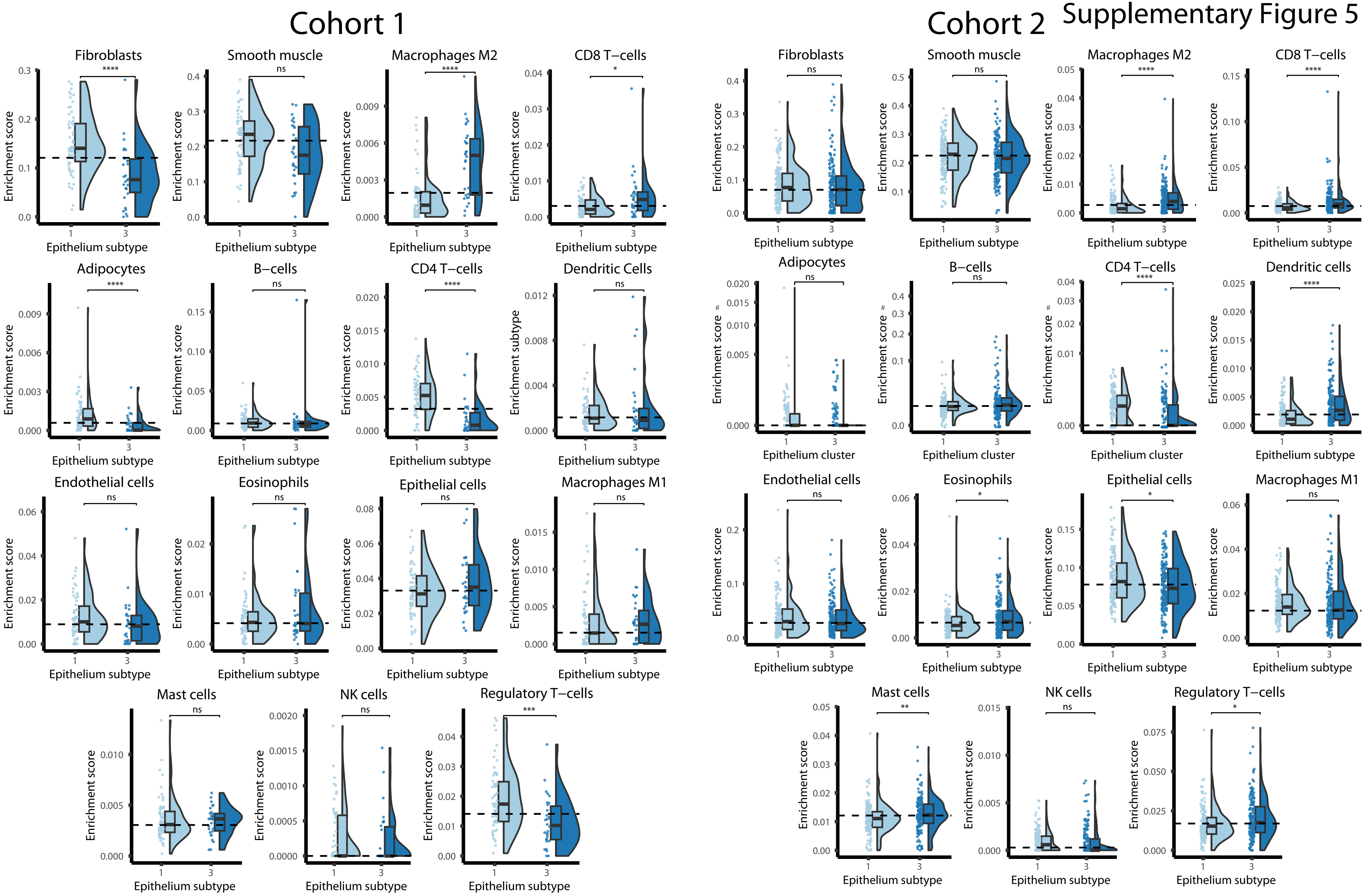

Supplementary Figure 5. Cell type enrichment analysis across epithelium subtype. Raincloud plots of enrichment scores for multiple cell types in E1 and E3 in cohort 1 and cohort 2. Wilcoxon rank-sum test used to determine significant differences between subtype E1 and E3. FDR corrected *p* values are reported. * = $p < 0.05$, ** = $p < 0.01$, *** = $p < 0.001$, **** = $p < 0.0001$. # = axis is square root scaled.

Supplementary Figure 6

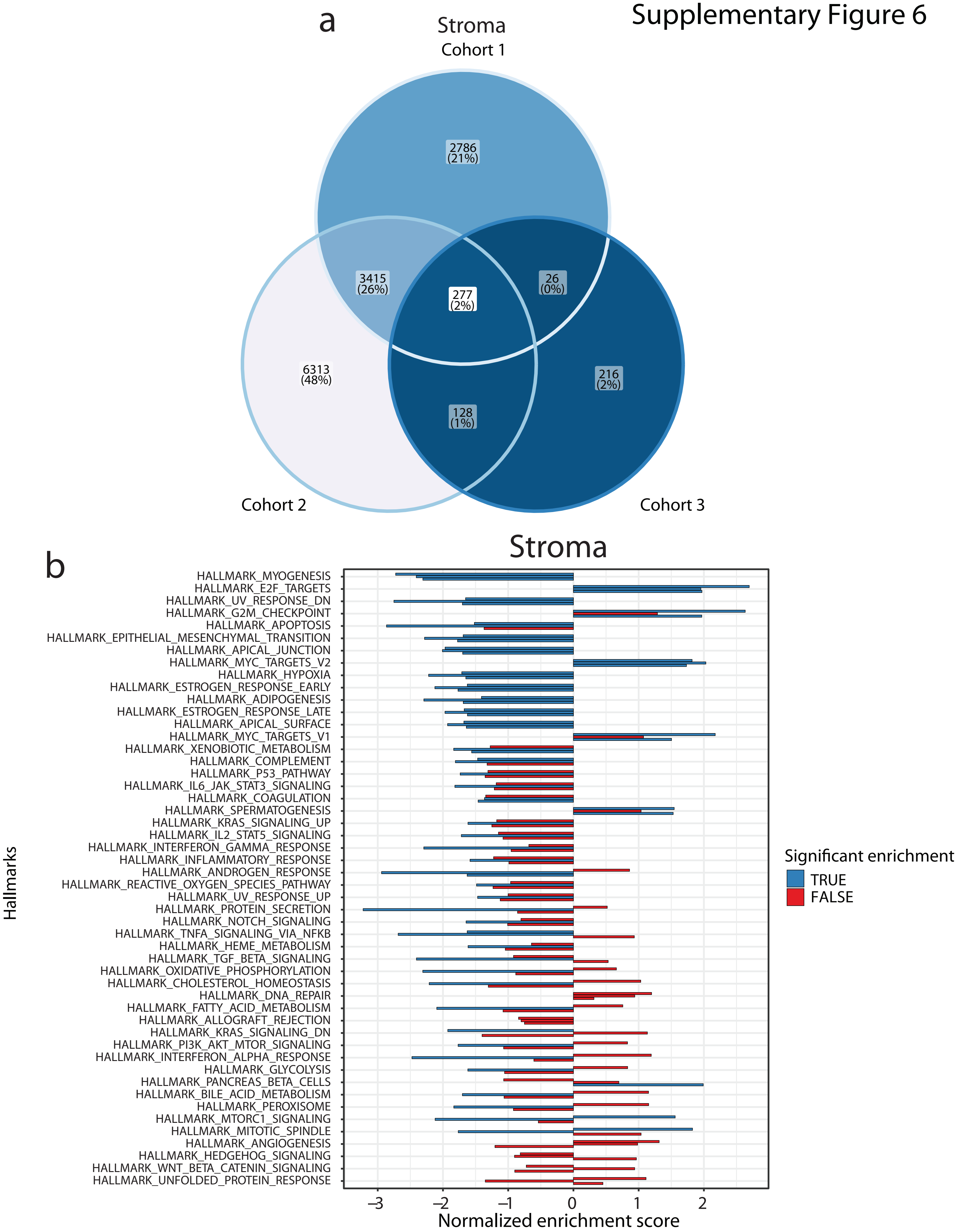

Supplementary Figure 6. Differential expression and Hallmark gene set enrichment analysis of stromal subtypes S1 and S3. (**a**) Venn diagram showing overlap of differentially expressed genes between S1 and S3 across cohorts. (**b**) Bar plot of normalized enrichment score and significance of Hallmark gene sets in GSEA of differentially expressed genes between S1 and S3. Significance based on FDR corrected *p* value.

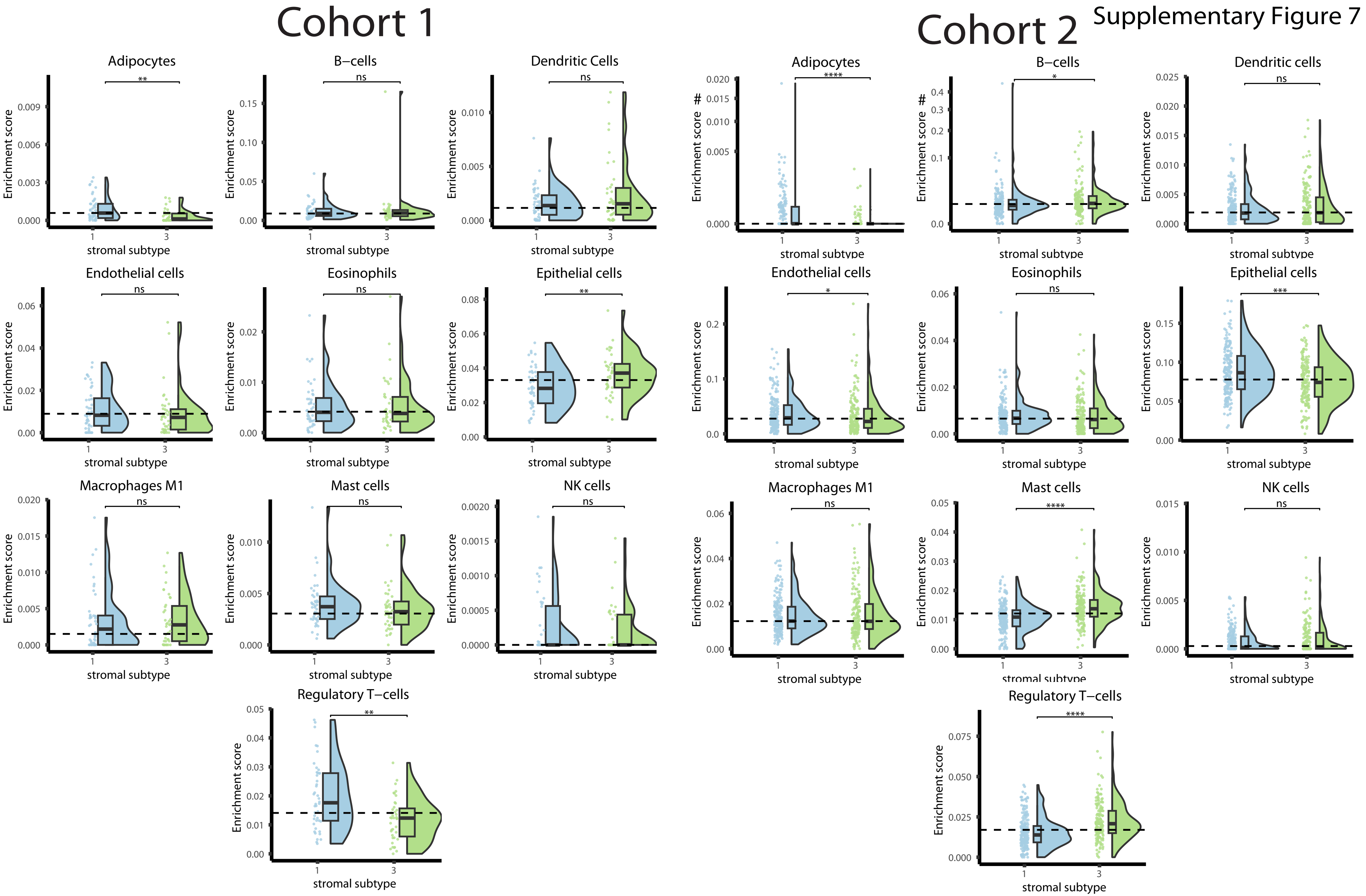

Suplementary Figure 7. Cell type enrichment analysis across stromal subtypes. Raincloud plots of enrichment scores for multiple cell types in S1 and S3 in cohort 1 and cohort 2. Wilcoxon rank-sum test used to determine significant differences between subtypes S1 and S3. FDR corrected p values are reported. * = $p < 0.05$, ** = $p < 0.01$, *** = $p < 0.001$, **** = $p < 0.0001$. # = axis is square root scaled.

Supplementary Figure 8

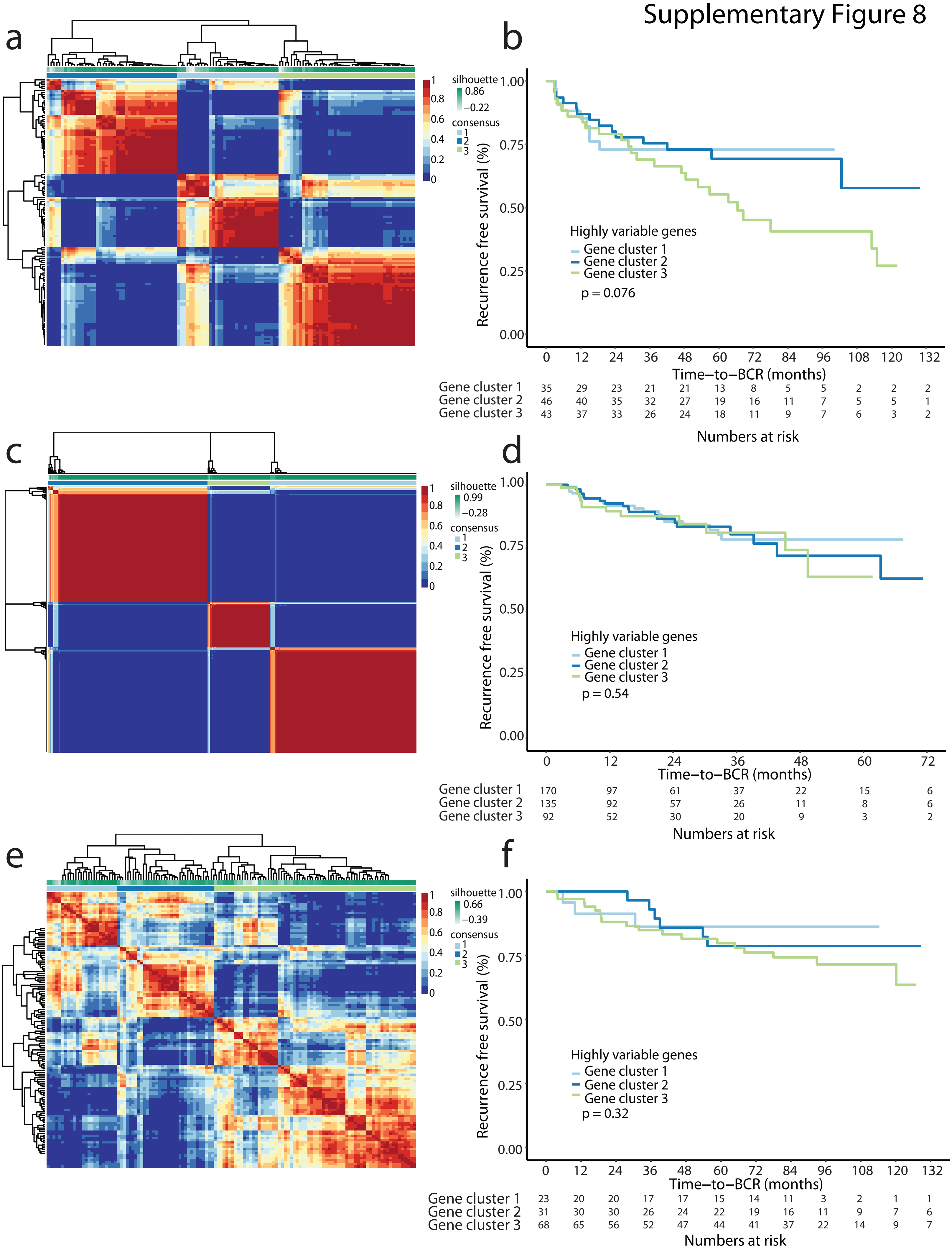

Supplementary Figure 8. Consensus heatmaps and Kaplan-Meier analysis of BCR risk inclustering of highly variable genes. Heatmap of NMF consensus clustering results for the top 500 most variable genes (based on standard deviation) in (**a**) cohort 1, (**c**) cohort 2, and (**e**) cohort 3. Kaplan-Meier plots for risk of BCR for each cluster from NMF consensus clustering of (**b**) cohort 1, (**d**) cohort 2, and (**f**) cohort 3. Significance determined using log-rank test.

Supplementary Figure 9

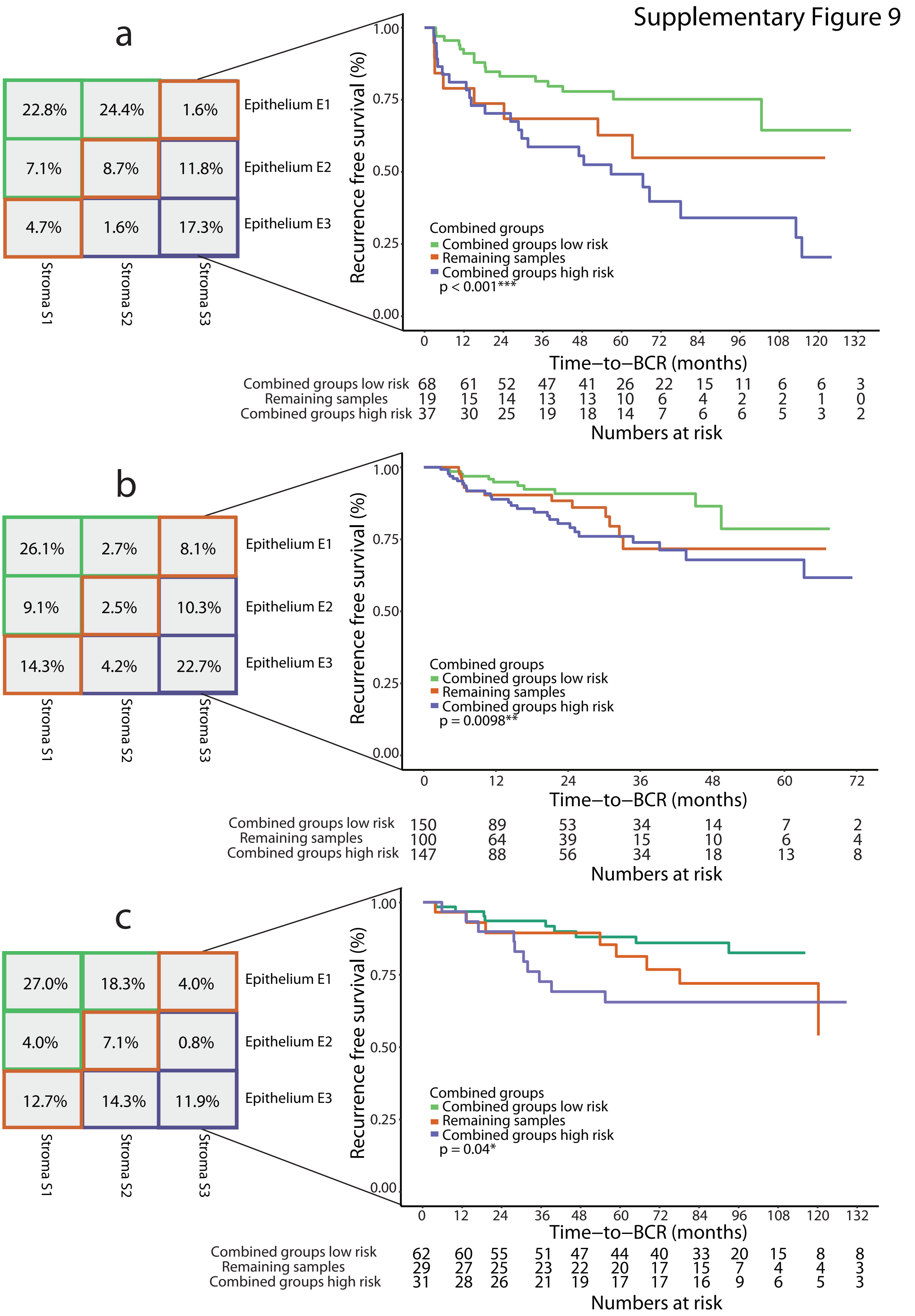

Supplementary Figure 9. Kaplan-Meier analysis of combined subtypes. Overlap in epithelial and stromal subtypes combined into groups of low risk or high risk and stratified in Kaplan-Meier analysis for cohort 1 (**a**), cohort 2 (**b**), and cohort 3 (**c**). Significance determined using log-rank test.

Supplementary Figure 10

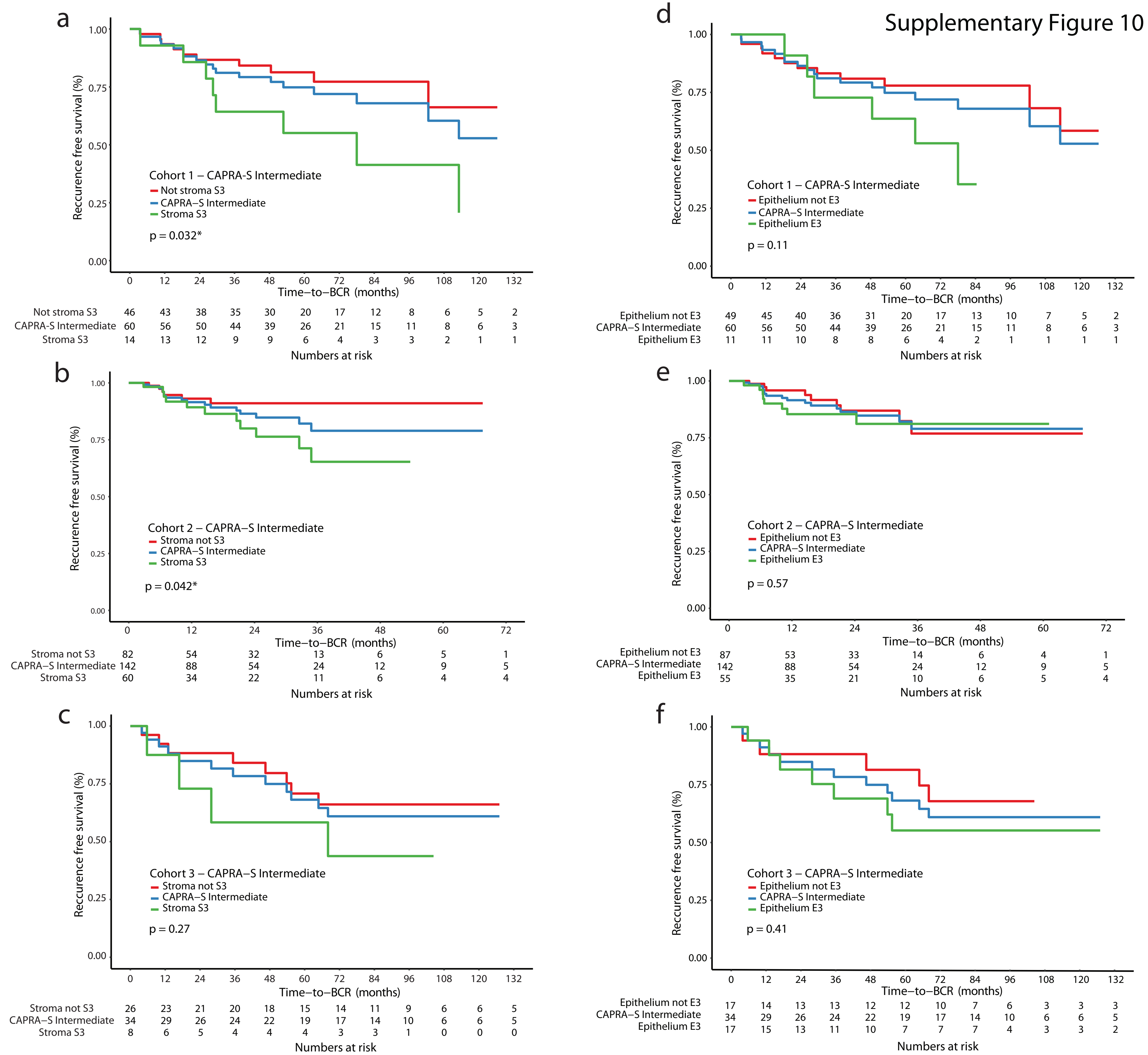

Supplementary Figure 10. Subtype separation of patients in the CAPRA-S intermediate risk group. Kaplan-Meier plot of BCR risk for CAPRA-S intermediate risk patents as one group or split in S3 and not S3 patients in (**a**) cohort 1, (**b**) cohort 2, and (**c**) cohort 3. CAPRA-S intermediate risk patents as one group or split in E3 and not E3 patients in (**d**) cohort 1, (**e**) cohort 2, and (**f**) cohort 3. Significance determined for S3 vs. not S3 patients, and E3 vs. not E3 patients, using log-rank test.

Supplementary Tables

Supplementary Table 1

Tissue	Symbol	ENSG
Epithelium	POTEG	ENSG00000222036
Epithelium	TMPRSS2	ENSG00000184012
Epithelium	ACPP	ENSG00000014257
Epithelium	AZGP1	ENSG00000160862
Epithelium	KLK2	ENSG00000167751
Epithelium	NPY	ENSG00000122585
Epithelium	SLC45A3	ENSG00000158715
Epithelium	DHCR24	ENSG00000116133
Epithelium	KLK3	ENSG00000142515
Epithelium	SORD	ENSG00000140263
Epithelium	RDH11	ENSG00000072042
Epithelium	TSPAN1	ENSG00000117472
Epithelium	CDH1	ENSG00000039068
Epithelium	NEFH	ENSG00000100285
Epithelium	KIAA1324	ENSG00000116299
Epithelium	ANPEP	ENSG00000166825
Epithelium	NKX3-1	ENSG00000167034
Epithelium	NEU1	ENSG00000204386
Epithelium	VEGFA	ENSG00000112715
Epithelium	KLK4	ENSG00000167749
Epithelium	SPDEF	ENSG00000124664
Epithelium	NCAPD3	ENSG00000151503
Epithelium	FOXA1	ENSG00000129514
Epithelium	DHRS7	ENSG00000100612
Epithelium	HGD	ENSG00000113924
Epithelium	GREB1	ENSG00000196208
Epithelium	CPNE4	ENSG00000196353
Epithelium	FOLH1	ENSG00000086205
Epithelium	EHF	ENSG00000135373
Epithelium	PRSS8	ENSG00000052344
Epithelium	GMPR	ENSG00000137198
Epithelium	FOLH1B	ENSG00000134612
Epithelium	GOLM1	ENSG00000135052
Epithelium	TRPV6	ENSG00000165125
Epithelium	NIPAL3	ENSG00000001461
Epithelium	RBM47	ENSG00000163694
Epithelium	TMEM141	ENSG00000244187
Epithelium	CREB3L4	ENSG00000143578
Epithelium	DPP4	ENSG00000197635
Epithelium	PRAC1	ENSG00000159182
Epithelium	KRT8	ENSG00000170421
Epithelium	FASN	ENSG00000169710
Epithelium	GRHL2	ENSG00000083307
Epithelium	TACSTD2	ENSG00000184292
Epithelium	PCAT4	ENSG00000251321
Epithelium	SYT7	ENSG00000011347
Epithelium	FXYD3	ENSG00000089356
Epithelium	PLA2G2A	ENSG00000188257
Epithelium	CD9	ENSG00000010278
Epithelium	LMAN1L	ENSG00000140506
Epithelium	SLC30A4	ENSG00000104154
Epithelium	ANO7	ENSG00000146205
Epithelium	NEDD4L	ENSG00000049759
Epithelium	ABCC4	ENSG00000125257
Epithelium	MLPH	ENSG00000115648
Epithelium	CPLX3	ENSG00000213578
Epithelium	BCAM	ENSG00000187244
Epithelium	ZG16B	ENSG00000162078
Epithelium	LINC01296	ENSG00000225210
Epithelium	PMEPA1	ENSG00000124225
Epithelium	AQP3	ENSG00000165272
Epithelium	RCAN3	ENSG00000117602
Epithelium	HOXB13	ENSG00000159184
Epithelium	ARG2	ENSG00000081181
Epithelium	DBI	ENSG00000155368
Epithelium	SFN	ENSG00000175793
Epithelium	NDRG1	ENSG00000104419
Epithelium	HMGN2P46	ENSG00000179362
Epithelium	MUC12	ENSG00000205277
Epithelium	C1orf116	ENSG00000182795
Epithelium	RAB3B	ENSG00000169213
Epithelium	PAK1IP1	ENSG00000111845
Epithelium	PLEKHH1	ENSG00000054690
Epithelium	RAB25	ENSG00000132698
Epithelium	AMD1	ENSG00000123505
Epithelium	SERINC5	ENSG00000164300
Epithelium	SCD	ENSG00000099194
Epithelium	SNORA74A	ENSG00000200959
Epithelium	TPT1	ENSG00000133112

Epithelium	SNORA21	ENSG00000199293
Epithelium	CD38	ENSG00000004468
Epithelium	SPINT2	ENSG00000167642
Epithelium	ZNF761	ENSG00000160336
Epithelium	FLNB	ENSG00000136068
Epithelium	ZNF525	ENSG00000203326
Epithelium	STEAP2	ENSG00000157214

**Tissue**	**Symbol**	**ENSG**
Stroma	MYH11	ENSG00000133392
Stroma	ACTG2	ENSG00000163017
Stroma	CNN1	ENSG00000130176
Stroma	ACTA2	ENSG00000107796
Stroma	TPM2	ENSG00000198467
Stroma	MYLK	ENSG00000065534
Stroma	TAGLN	ENSG00000149591
Stroma	DES	ENSG00000175084
Stroma	PCP4	ENSG00000183036
Stroma	SPARCL1	ENSG00000152583
Stroma	LMOD1	ENSG00000163431
Stroma	MGP	ENSG00000111341
Stroma	MYL9	ENSG00000101335
Stroma	SYNPO2	ENSG00000172403
Stroma	SORBS1	ENSG00000095637
Stroma	NEXN	ENSG00000162614
Stroma	PGM5	ENSG00000154330
Stroma	CALD1	ENSG00000122786
Stroma	SYNM	ENSG00000182253
Stroma	DCN	ENSG00000011465
Stroma	CSRP1	ENSG00000159176
Stroma	SPARC	ENSG00000113140
Stroma	CCDC80	ENSG00000091986
Stroma	HSPB8	ENSG00000152137
Stroma	COL6A3	ENSG00000163359
Stroma	TIMP2	ENSG00000035862
Stroma	TNS1	ENSG00000079308
Stroma	IGF1	ENSG00000017427
Stroma	C12orf75	ENSG00000235162
Stroma	C7	ENSG00000112936
Stroma	RBPMS	ENSG00000157110
Stroma	FLNC	ENSG00000128591
Stroma	ACTC1	ENSG00000159251
Stroma	MAP1B	ENSG00000131711
Stroma	ATP2B4	ENSG00000058668
Stroma	CAV1	ENSG00000105974
Stroma	PDE5A	ENSG00000138735
Stroma	SVIL	ENSG00000197321
Stroma	COL1A2	ENSG00000164692
Stroma	AOC3	ENSG00000131471
Stroma	ACTA2-AS1	ENSG00000180139
Stroma	RGS2	ENSG00000116741
Stroma	TPM1	ENSG00000140416
Stroma	NID1	ENSG00000116962
Stroma	OGN	ENSG00000106809
Stroma	PRELP	ENSG00000188783
Stroma	EDNRA	ENSG00000151617
Stroma	MYADM	ENSG00000179820
Stroma	PDLIM3	ENSG00000154553
Stroma	CLU	ENSG00000120885
Stroma	FN1	ENSG00000115414
Stroma	C1S	ENSG00000182326
Stroma	ALDH1A1	ENSG00000165092
Stroma	SMOC1	ENSG00000198732
Stroma	DDR2	ENSG00000162733
Stroma	KANK2	ENSG00000197256
Stroma	CDC42EP3	ENSG00000163171
Stroma	SERPING1	ENSG00000149131
Stroma	AEBP1	ENSG00000106624
Stroma	GSN	ENSG00000148180
Stroma	MSN	ENSG00000147065
Stroma	LGALS1	ENSG00000100097
Stroma	MYOCD	ENSG00000141052
Stroma	FERMT2	ENSG00000073712
Stroma	VCL	ENSG00000035403
Stroma	TUBA1A	ENSG00000167552
Stroma	A2M	ENSG00000175899
Stroma	INF2	ENSG00000203485
Stroma	MEIS1	ENSG00000143995
Stroma	HSPB6	ENSG00000004776
Stroma	PDLIM7	ENSG00000196923
Stroma	NR2F1	ENSG00000175745
Stroma	CCND2	ENSG00000118971
Stroma	COL1A1	ENSG00000108821
Stroma	LAMA4	ENSG00000112769
Stroma	IGFBP7	ENSG00000163453
Stroma	MFAP4	ENSG00000166482
Stroma	CRYAB	ENSG00000109846

Stroma	HSPG2	ENSG00000142798	Stroma	CNTN1	ENSG00000018236
Stroma	LUM	ENSG00000139329	Stroma	COL14A1	ENSG00000187955
Stroma	TIMP3	ENSG00000100234	Stroma	FBLN1	ENSG00000077942
Stroma	SLC8A1	ENSG00000183023	Stroma	ATP1A2	ENSG00000018625
Stroma	PTGIS	ENSG00000124212	Stroma	PALLD	ENSG00000129116

Supplementary Table 1. Gene list of epithelium-specific signature and stroma-specific signature.

Supplementary Table 2			
Stroma subtype S1 feature genes		Stroma subtype S3 feature genes	
**ENSG**	**Symbol**	**ENSG**	**Symbol**
ENSG00000112936	C7	ENSG00000165092	ALDH1A1
ENSG00000175899	A2M	ENSG00000108821	COL1A1
ENSG00000139329	LUM	ENSG00000115414	FN1
ENSG00000011465	DCN	ENSG00000111341	MGP
ENSG00000187955	COL14A1	ENSG00000164692	COL1A2
ENSG00000017427	IGF1	ENSG00000113140	SPARC
ENSG00000091986	CCDC80	ENSG00000122786	CALD1

Supplementary Table 2. Gene list of stroma-specific signature genes important for placement into stromal subtype S1 and S3.

Supplementary Table 3

Variable	Cohort 1 N	HR	95% CI	*p*-value	Cohort 2 N	HR	95% CI	*p*-value	Cohort 3 N	HR	95% CI	*p*-value
	Univariate cox regression											
**Stroma**												
Intermediate-S1/S2	46	-	-	-	82	-	-	-	26	-	-	-
CAPRA-S Intermediate	60	1.35	0.65 - 2.85	0.4	142	1.69	0.67 - 4.29	0.3	34	1.20	0.49 - 2.93	0.7
Intermediate-S3	14	2.66	1.06 - 6.67	**0.036**	60	2.71	1.00 - 7.33	**0.049**	8	1.97	0.59 - 6.55	0.27
**Epithelium**												
Intermediate-E1/E2	49	-	-	-	87	-	-	-	17	-	-	-
CAPRA-S Intermediate	60	1.21	0.60 - 2.45	0.6	142	1.13	0.50 - 2.53	0.8	34	1.29	0.45 - 3.65	0.6
Intermediate-E3	11	2.20	083 - 5.84	0.1	55	1.31	0.51 - 3.40	0.6	11	1.62	0.51 - 5.11	0.4

Supplementary Table 2. Univariate Cox regression analysis using stromal subtypes to re-stratify CAPRA-S intermediate risk.
